# Combination of KRAS ASO and RIG-I agonist in extracellular vesicles transforms the tumor microenvironment towards effective treatment of KRAS-dependent cancers

**DOI:** 10.7150/thno.105519

**Published:** 2025-06-09

**Authors:** Cao Dai Phung, Trinh T.T. Tran, Brendon Zhi Jie Yeo, Rebecca Carissa Prajogo, Evelyn Saudjana, Eric Yew Meng Yeo, Chang Gao, Phuong H.D. Nguyen, Migara Kavishka Jayasinghe, Xuan T.T. Dang, Celest Phang Lixuan, Trinh Mai Nguyen, Boya Peng, Anh Hong Le, Tram T.T Nguyen, Gloria Mei En Chan, Yuin-Han Loh, Boon Cher Goh, Wai Leong Tam, Glenn Kunnath Bonney, Dahai Luo, Minh T.N. Le

**Affiliations:** 1 Department of Pharmacology and Institute for Digital Medicine, Yong Loo Lin School of Medicine, National University of Singapore, Singapore.; 2 Cancer Science Institute of Singapore, National University of Singapore, Singapore.; 3 Genome Institute of Singapore, A*STAR, Singapore.; 4 Lee Kong Chian School of Medicine, Nanyang Technological University, Singapore.; 5 Institute of Structural Biology, Nanyang Technological University, Singapore.; 6 Synthetic Biology Translational Research Program, Yong Loo Lin School of Medicine, National University of Singapore, Singapore.; 7 Cell Fate Engineering and Therapeutics Laboratory, A*STAR Institute of Molecular and Cell Biology, Singapore, 138673, Singapore.; 8 Department of Biological Sciences, National University of Singapore, Singapore, 117543, Singapore.; 9 Integrative Sciences and Engineering Programme (ISEP), NUS Graduate School, National University of Singapore, 21 Lower Kent Ridge Road, Singapore, 119077, Singapore.; 10 Department of Physiology, Yong Loo Lin School of Medicine, National University of Singapore, Singapore, 117593, Singapore.; 11 Department of Surgery, Yong Loo Lin School of Medicine, National University of Singapore.; 12 Institute of Molecular and Cell Biology, A*STAR, Singapore.; 13 Vingroup Science and Technology Scholarship Program, Vin University, Hanoi, Vietnam.; 14 Nanomedicine Translational Research Programme, Yong Loo Lin School of Medicine, National University of Singapore, Singapore.; 15 Cancer Translational Research Programme, Yong Loo Lin School of Medicine, National University of Singapore, Singapore.; 16 Institute of Health Innovation & Technology, National University of Singapore, Singapore.

**Keywords:** extracellular vesicles, antisense oligonucleotides, RIG-I agonist, KRAS mutation, cancer therapy

## Abstract

**Rationale**: Mutations in the *KRAS* gene drive many cancers, yet targeting *KRAS* mutants remains a challenge. Here, we address this hurdle by utilizing a nucleic acid-based therapeutic strategy delivered via extracellular vesicles (EVs) to simultaneously inhibit *KRAS* mutants and activate the RIG-I pathway, aiming to enhance anti-tumor immunity.

**Methods**: Antisense oligonucleotides against *KRAS* mutants (*KRAS* ASOs) and RIG-I agonist immunomodulatory RNA (immRNA) were loaded into EVs and administered to *KRAS*-mutant cancer models. The therapeutic effects were assessed in colorectal and non-small cell lung cancer (NSCLC) tumor models, as well as patient-derived pancreatic cancer organoids. Immune responses were evaluated by analyzing tumor microenvironment's changes, dendritic cell activation, and T cell memory formation. The treatment efficacy was evaluated based on the tumor development and overall survival.

**Results**: The KRAS-ASO and immRNA combination treatment induced immunogenic tumor cell death and upregulated interferons in *KRAS*-dependent cancers. In a colorectal tumor model, the therapy shifted the tumor microenvironment to an immunogenic state, activated dendritic cells in sentinel lymph nodes, and promoted memory T cell formation. In an aggressive NSCLC model, the treatment resulted in a strong anti-tumor activity and extended survival without any adverse effects. Validation in patient-derived pancreatic cancer organoids confirmed the clinical translation potential of this approach.

**Conclusions**: EV-mediated delivery of ASOs and immRNA effectively inhibits *KRAS* mutants and activates RIG-I, leading to a robust anti-tumor immune response. This strategy holds promise for effectively treating *KRAS*-driven cancers and improving clinical outcomes.

## Introduction

Mutation in the Kirsten rat sarcoma virus (*KRAS*) gene, a member of the RAS family, has been reported to be an oncogenic driver in multiple solid tumor types, including pancreatic cancer, colorectal cancer, and lung cancer 1. *KRAS* gene encodes KRAS protein, a GTPase functioning as a molecular switch by converting between activated GTP-bound and inactivated GDP-bound states 2. In cancer, point mutations in the *KRAS* gene disrupt this cycle, keeping the KRAS protein in a constitutively activated GTP-bound state that drives tumorigenesis, while in normal cells, KRAS protein is predominantly in the GDP-bound state 3. Notably, the activation of oncogenic KRAS mutants leads to the inactivation of various tumor suppressor pathways in cancer, such as TP53, INK4a-ARF, and DPC4-SMAD4 4. Clinical evidence from patients with aggressive clinical phenotypes in cancer patients shows that KRAS mutations lead to poor therapeutic outcomes and overall survival, regardless of cancer stage, emphasizing the need for targeted therapy for KRAS-mutated cancers 5,6.

In addition to promoting cancer cell survival and proliferation, KRAS mutants also favor the formation of a highly immunosuppressive tumor microenvironment (TME) through several mechanisms, including repressing interferon regulatory factors (IRFs) 7,8, increased secretion of immunosuppressive cytokines (i.e., TGF-β and IL-10) 9, activating the JAK-STAT3 signaling pathway 10, triggering the downregulation of MHC-I in tumor cells 11, and synergizing with other mutations, such as mutations in the *TP53* gene 12. Despite considerable therapeutic efforts, successful strategies for targeting KRAS mutations have remained elusive, except for the recent breakthrough with the development of KRAS G12C inhibitor, due to the lack of accessible pockets that allow small molecules to bind with high affinity 13. While KRAS G12C inhibitors have demonstrated clinical efficacy, concerns have been raised regarding the emergence of acquired drug resistance 14. Cancer cells can limit the efficacy of KRAS inhibitors through several mechanisms, such as activating other signaling pathways, developing new resistance mutations, or downregulating multiple immune signals associated with the activation of anti-cancer immune responses 15. Therefore, novel approaches such as combination therapies are necessary to improve clinical outcomes in patients with KRAS-mutated tumors.

Antisense oligonucleotides (ASOs) are short, single-stranded nucleic acids designed to modulate gene expression by promoting RNase H-mediated RNA degradation or altering RNA splicing 16. By designing ASOs to target specific mutations in diseased cells, their therapeutic action can be highly selective, minimizing off-target effects and reducing toxicity to normal tissues. This specificity also positions ASOs as an integral component of personalized medicine, allowing for individualized therapeutic designs tailored to the genetic profile of each patient 17. To date, the U.S. Food and Drug Administration (FDA) and the European Medicines Agency (EMA) have approved 16 ASOs for treating diverse diseases, including retinal disorders, blood-related conditions, neuromuscular diseases, and cardiovascular ailments 18. ASOs targeting KRAS have shown promise in several studies, demonstrating efficacy in suppressing tumor growth 19-21. However, current KRAS-targeting ASOs are predominantly pan-KRAS inhibitors, which do not distinguish between the mutants from wild-type (WT) KRAS. This lack of specificity raises concerns about potential toxicity to normal cells, thereby limiting their therapeutic index.

A critical feature of inflamed tumors is the high level of type-I interferons (IFNs) that contribute to the spontaneous induction of anti-tumor T cell responses. An emerging approach to promote IFN-mediated proinflammatory condition within the TME is to activate retinoic acid-inducible gene I (RIG-I), a cytoplasmic molecule ubiquitously expressed in human cells 22. The RIG-I pathway is activated when RNA binds to the 5′-triphosphate end of the carboxy-terminal domain (CTD) or the helicase domain of RIG-I, leading to the activation of various downstream factors, including IRF3, IRF7, AP1, and NF-κB that subsequently upregulates the expression of *IFN* genes 23. The activation of the RIG-I pathway can induce proapoptotic signaling pathways in cancer cells. Notably, normal cells are much less susceptible to apoptosis mediated by RIG-I activation than tumor cells, as they can upregulate or activate specifically Bcl-x_L_ to counteract the proapoptotic effect 24-26. This safety mechanism seems to be deficient in malignant cells that have undergone numerous genetic alterations, making RIG-I activation-mediated apoptosis specific to tumor cells while limited in non-malignant cells. Mechanistically, oncogenic KRAS mutations contribute to the suppression of IRF-mediated signaling 7,8, leading to a dampened IFN response and allowing tumors to evade immune surveillance. Notably, clinical studies revealed that the KRAS G12C inhibitor treatment can robustly induce the production of IFNs in the tumor 7,27.

In a previous study, we demonstrated the potent anti-cancer activity and the safety of an immunomodulatory RNA (immRNA), a novel RIG-I agonist composed of a hairpin RNA molecule with 5' triphosphate, delivered by red blood cell-derived extracellular vesicles (RBCEVs) for the treatment of primary tumors and lung metastasis of breast cancer 26. Given the immunosuppressive effects of KRAS mutations and their potential role in limiting RIG-I pathway activation, combining KRAS ASO with immRNA represents a rational therapeutic approach. This combination therapy aims to restore IFN signaling suppressed by oncogenic KRAS while simultaneously promoting proinflammatory conditions in the TME, ultimately leading to enhanced tumor cell death and immune activation. Consequently, this combination regimen may overcome acquired resistance associated with the treatment of KRAS inhibitors.

Here, we address the challenges of limited therapeutic efficacy in aggressive KRAS-mutated cancers through the deployment of nucleic acid-based therapeutics. We describe an approach to robustly inflame the TME of KRAS-dependent cancers by combining an ASO against the mutated *KRAS* (*KRAS* ASO) and immRNA to simultaneously suppress the *KRAS* mutant and activate the RIG-I pathway in tumor cells, respectively. We utilize RBCEVs to deliver these nucleic acids to the tumor since RBCEVs are a biocompatible carrier of nucleic acids that can be harnessed in a scalable manner as we have shown in our previous studies 26,28-30. We demonstrate that RBCEVs loaded with ASOs efficiently inhibit mutated *KRAS* genes while sparing cells with the WT gene, leading to tumor cell death. Importantly, the combination of *KRAS* ASO and immRNA delivered by RBCEVs induced synergistic activation of RIG-I, leading to robust induction of tumor cell death and upregulation of IFNs in KRAS-dependent cancers both *in vitro* and *in vivo*. Of note, this combination treatment effectively shifts the “cold” TME with *KRAS* mutation into a “hot” environment, leading to the induction of potent anti-tumor activity without any observable adverse effects in both a colorectal cancer model and an aggressive non-small cell lung cancer (NSCLC) model. Furthermore, we validate the anti-cancer activity of this approach in patient-derived pancreatic cancer organoids with the *KRAS* G12D mutation, demonstrating the clinical translation potential of our approach for the treatment of KRAS-addicted cancers.

## Results

### RBCEVs efficiently deliver small nucleic acids to *KRAS*-mutated tumor cells

**Figure 1:**
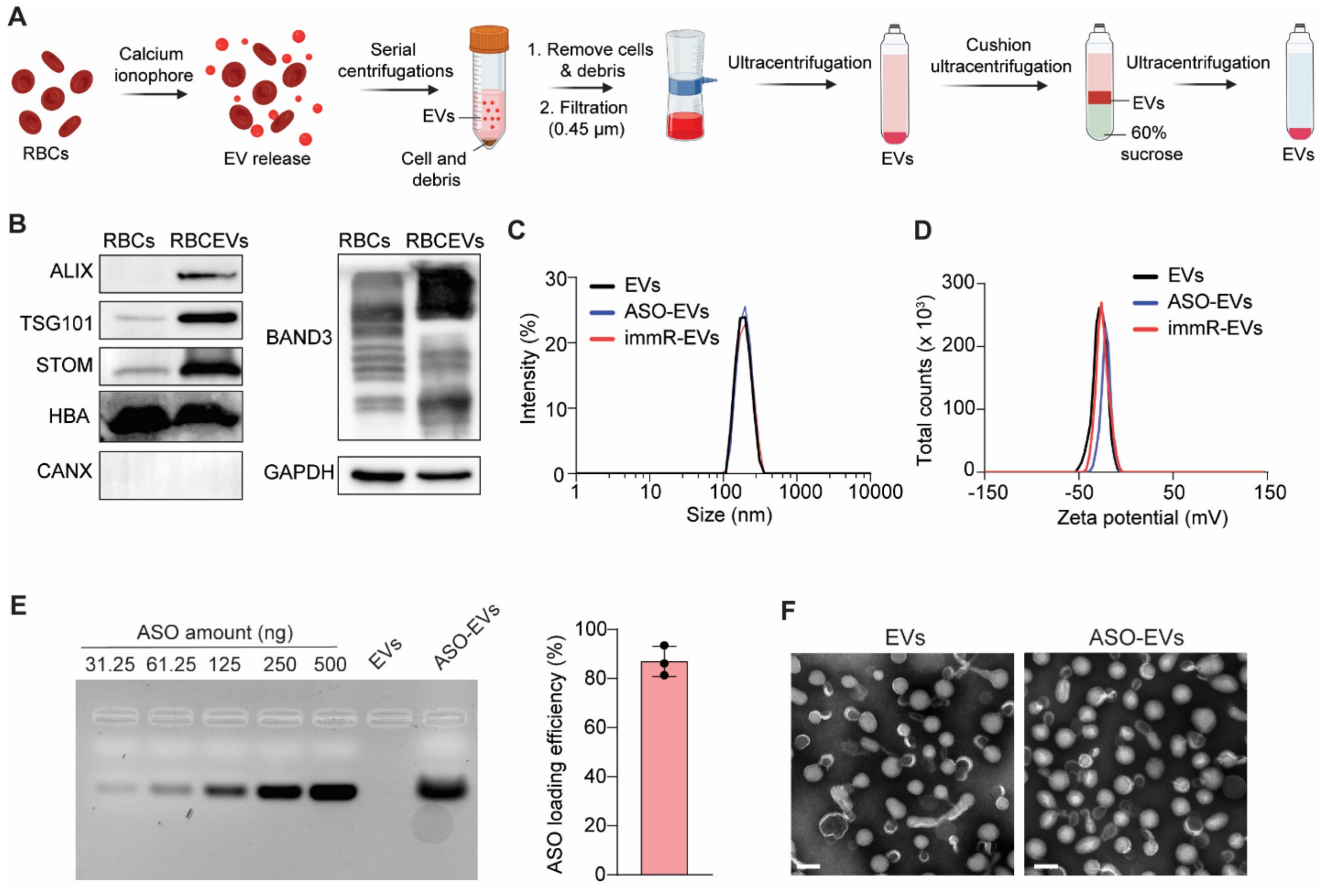
** RBCEVs can be efficiently loaded with small nucleic acids for delivery to KRAS-mutated tumor cells.** (**A**) Schematic for the RBCEV purification process from red blood cells (RBCs). (**B**) Western blot analysis of proteins in RBCs and purified RBCEVs. (**C**-**D**) Dynamic light scattering analysis of (**C**) size distribution and (**D**) zeta potential of unloaded EVs, ASO-loaded EVs (ASO-EVs), and immRNA-loaded EVs (immR-EVs). (**E**) Loading efficiency of NC ASO in RBCEVs using REG1 transfection reagent determined by gel electrophoresis (n=3). (**F**) Representative transmission electron microscopy images of RBCEVs and NC-ASO-loaded RBCEVs. Scale bar: 100 nm.

We purified RBCEVs according to our previously established protocol (Figure 1A) 28. Western blot analysis (Figure 1B) showed that the purified RBCEVs were enriched in typical EV markers, such as ALIX and TSG101, as well as RBCEV-specific markers, Stomatin (STOM). Notably, Calnexin (CANX), the endoplasmic reticulum marker, which is commonly found in various cell types, was absent in RBCEVs. Furthermore, RBCEVs are also highly expressed hemoglobin A (HBA) and Band3, the major RBC proteins. These data confirm the identity and purity of isolated RBCEVs, with no evidence of contamination from other blood EV types. We then loaded ASO or immRNA into RBCEVs using REG-1 transfection reagent as previously reported 26. We observed that the loading of ASO or immRNA did not significantly alter the particle size and polydispersity index (PDI) of loaded EVs (Figure 1C). However, it slightly increased their zeta potential, as analyzed by dynamic light scattering (Figure 1D). The loading efficiency of was subsequently quantified using agarose gel electrophoresis. As shown in Figure 1E, the ASOs were efficiently loaded into RBCEVs using the REG-1 reagent. Transmission electron microscopy (TEM) revealed that after being loaded with ASO, RBCEVs maintained a structural morphology similar to that of the unloaded control (Figure 1F).

The stability of RBCEVs was evaluated by assessing their sizes, zeta potential, and concentrations after incubation in various conditions, including 4% trehalose (storage buffer), PBS pH 7.4 (physiological pH), 50 mg/mL BSA in PBS (mimicking blood protein concentration), and PBS pH 6.0 (the acidic condition of the TME). Across all tested conditions, no significant changes were observed in these parameters (Figure S1A-C), demonstrating the high stability of RBCEVs as a robust delivery system. Next, we evaluated the stability of ASO and immRNA in the storage buffer (4% trehalose in PBS, pH 7.4) at -80 °C using agarose gel electrophoresis of ASO- or immRNA-loaded EVs at different time points. As shown in Figure S1D-G, immRNA remained stable in 4% trehalose at -80 °C for up to 24 h, whereas ASO exhibited stability for up to 96 h.

We then treated *KRAS* mutant A427 cells with different doses of RBCEVs to evaluate their potential toxicity using cell counting kit-8 (CCK-8) assay. The results demonstrated that RBCEVs were non-toxic to the treated cells, even at a high dose of 1 mg/mL (Figure S2). We further investigated the uptake of RBCEVs loaded with Alexa Fluor 488 (AF488)-labeled ASO by mutated *KRAS*-harboring cancer cells, including A427 cells (*KRAS* G12D) and H441 cells (*KRAS* G12V) (Figure S3A), using flow cytometry. We observed that the RBCEVs could efficiently deliver the ASO into these cancer cells only after a short incubation time (2 h) and at a low RBCEV concentration (5 µg/µL) (Figure S3B), suggesting that RBCEVs are a suitable delivery vehicle for small nucleic acid drugs to these cancer cells.

We then designed several 20-nucleotide gapmer ASOs to specifically target *KRAS* G12D and *KRAS* G12V mutations, while sparing WT *KRAS* gene. The ASOs were chemically modified with locked nucleic acid (LNA), phosphorothioate (PS), and 2'-O-methoxyethyl (2'-MOE) to increase the stability and specificity of the ASOs (Figure S4A). We then tested these ASOs in *KRAS* mutant-bearing cancer cell lines, including A427 (*KRAS* G12D) and H441 (*KRAS* G12V) human lung cancer cells, to examine the specific knockdown of *KRAS* by the ASO-loaded EVs. Human lung H1975 cells, which harbor the WT *KRAS* gene, were used as a control cell line. By Western blot analysis, we found that *KRAS* G12D ASO2 and *KRAS* G12V ASO2 showed significant knockdown efficacy of the corresponding mutant gene in A427 and H441 cells (Figure S4B, and S4D). Consequently, there was a noticeably reduced viability of mutant-bearing lung cancer cells, as determined by CCK-8 assay, while minimizing the cross-reaction in WT *KRAS* gene-harboring H1975 cells (Figure S4C and S4E).

Our approach represents a significant innovation as it allows for precise discrimination between mutant and WT KRAS, a challenge in developing KRAS inhibitors for cancer therapy. This selective inhibition highlights the potential of these ASOs as a novel and highly targeted therapeutic strategy to address a critical unmet need in the treatment of KRAS-driven cancers. These results also indicated that RBCEVs represent a suitable platform for delivering ASOs to cancer cells and that deploying oncogene-specific ASOs may be a promising approach for cancer treatment, due to the highly specific inhibition of driver mutations in cancer cells while sparing WT genes in normal cells.

### Combination of KRAS mutation-targeting ASO with RIG-I agonist RNA synergistically potentiates the RIG-I pathway activation in KRAS-driven cancer cells

**Figure 2:**
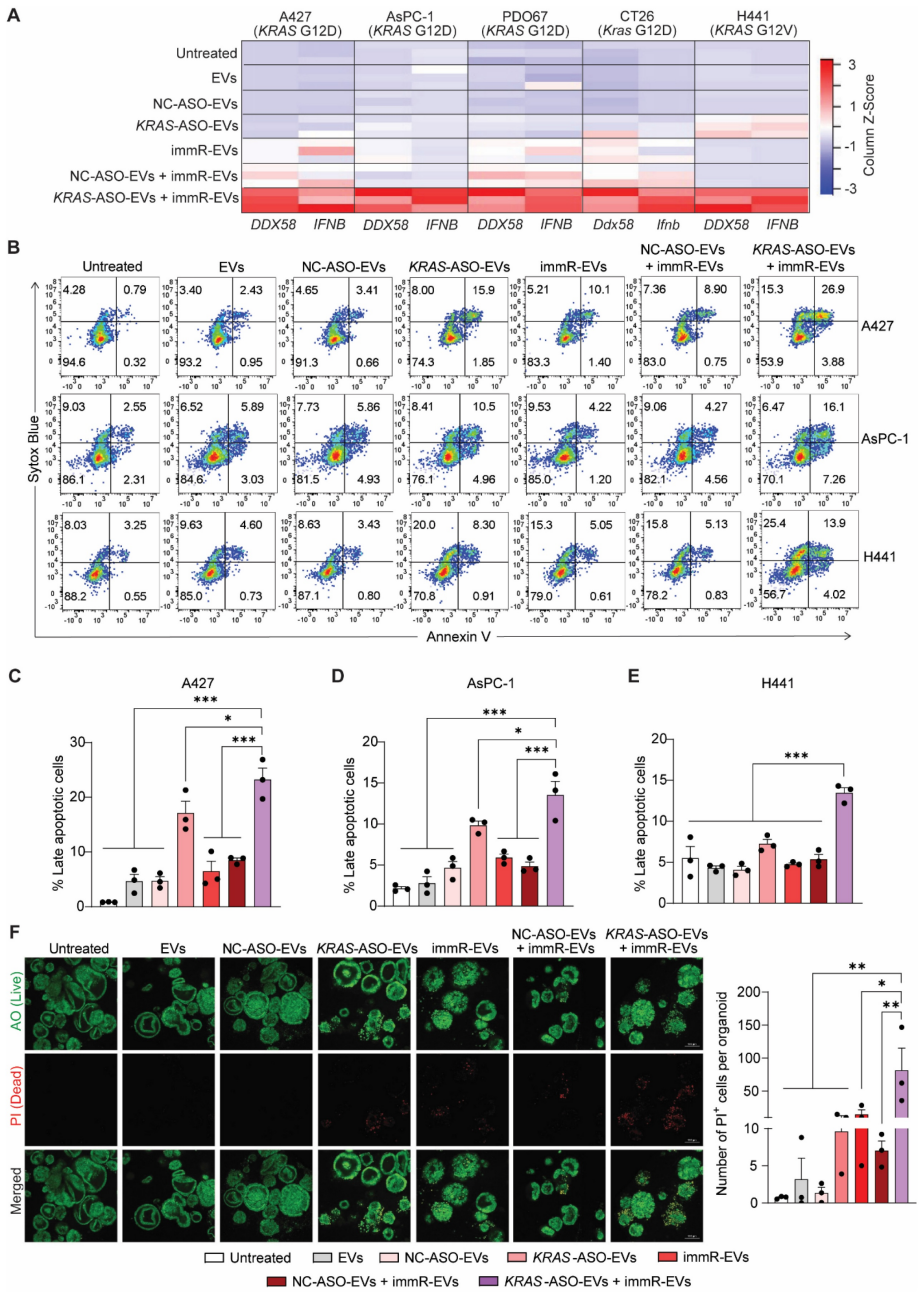
** Combination treatment of ASOs targeting *KRAS* mutations with RIG-I agonist immunomodulatory RNA in RBCEVs synergistically activates the RIG-I signaling pathway and induces apoptosis in KRAS-mutated cancer cells.** (**A**) Heatmap visualization of *DDX58* and *IFNB* expression in A427 lung cancer cells (*KRAS* G12D), AsPC-1 pancreatic cancer cells (*KRAS* G12D), PDO67 patient-derived pancreatic cancer organoids (*KRAS* G12D), CT26 colorectal cancer cells (*Kras* G12D), and H441 lung cancer cells (*KRAS* G12V) after treatments with 25 µg/mL RBCEVs or RBCEVs loaded with either NC ASO (NC-ASO-EVs, 50 µg/mL EVs), *KRAS* ASO (*KRAS*-ASO-EVs, 25 µg/mL EVs), immRNA (immR-EVs, µg/mL EVs), combined NC ASO and immRNA (NC-ASO-EVs + immR-EVs, 50 µg/mL EVs), or combined *KRAS* ASO and immRNA (*KRAS*-ASO-EVs + immR-EVs, 50 µg/mL EVs) for 48 h. Z-Score was computed using the formula: (Gene expression value - mean expression across all samples) / Standard Deviation. The Gplots package in R software was used to plot a heatmap based on the computed Z-score. (**B**) Flow cytometric analysis of Annexin V/SYTOX Blue staining in A427, AsPC-1, and H441 cells 24 h post-treatment with different RBCEV formulations. (**C**-**E**) Proportion of late apoptosis (Annexin^+^, SYTOX Blue^+^) in (**C**) A427, (**D**) AsPC-1, and (**E**) H441 cells which were treated as described in (B) (n = 3). (**F**) Assessment of apoptosis in PDO67 patient-derived pancreatic cancer organoids after treatments with different RBCEV formulations for 72 h by staining the treated organoids with acridine orange (AO) and propidium iodide (PI). The graphs present mean ± SEM. *P < 0.05, **P < 0.01, and ***P < 0.001, determined using One-Way ANOVA test.

KRAS mutations have been reported to drive immune evasion in several types of cancer and evidence from both pre-clinical and clinical settings has demonstrated that KRAS inhibitors can activate pro-inflammatory cytokine signaling in cancer cells 31. We thus investigated the anti-cancer potency of the combinatorial regime of *KRAS* ASO and immRNA delivered by RBCEVs to various *KRAS* mutation-bearing cancer cell types, including A427 lung cancer cells (*KRAS* G12D), AsPC-1 pancreatic cancer cells (*KRAS* G12D), PDO67 patient-derived pancreatic cancer cells (*KRAS* G12D), CT26 colorectal cancer cells (*Kras* G12D), and H441 lung cancer cells (*KRAS* G12V). We first quantified the expression level of two important genes in the RIG-I pathway, including *DDX58* and *IFNB*, in EV-treated cells using qPCR. Interestingly, the cells that received the *KRAS* ASO and immRNA combination treatment exhibited the highest expression of both *DDX58* and *IFNB* compared to those receiving the single treatments or control treatments (Figure 2A & Figure S5A-D). It is noted that we found a similar observation when the AsPC-1 cells were treated with ASO and immRNA delivered by lipid nanoparticles (LNPs) (Figure S5E-G). Additionally, apoptosis assays revealed that the combination treatment significantly increased apoptosis in all tested KRAS-mutated cancer cells (Figure 2B-E). We then extended our validation to patient-derived pancreatic cancer organoids with *KRAS* G12D mutation by live/dead staining (Figure 2F). Under confocal microscope analysis, the treatment with *KRAS*-ASO-EVs or immR-EVs clearly induced cell death in the organoids as evidenced by the destabilization of organoid structure and observed fluorescence signal propidium iodide (PI) within the organoids. In agreement with the findings in cancer cell lines from 2D culture, combining immR-EVs with *KRAS*-ASO-EVs further increased the resultant anti-cancer potency, leading to the highest level of organoid destruction and PI signal detected. By Western blot analysis, we observed a reduced expression of the anti-apoptotic proteins MCL-1 and BCL-2, which are typically upregulated by mutant KRAS 32,33 as well as p-ERK1/2, a downstream effector of KRAS (Figure S6). Notably, this combination treatment showed no noticeable toxicity on primary human bronchial epithelial cells (HBECs), as demonstrated by Annexin-V/PI assay (Figure S7), demonstrating the clinically translational potential of our proposed approach.

**Figure 3:**
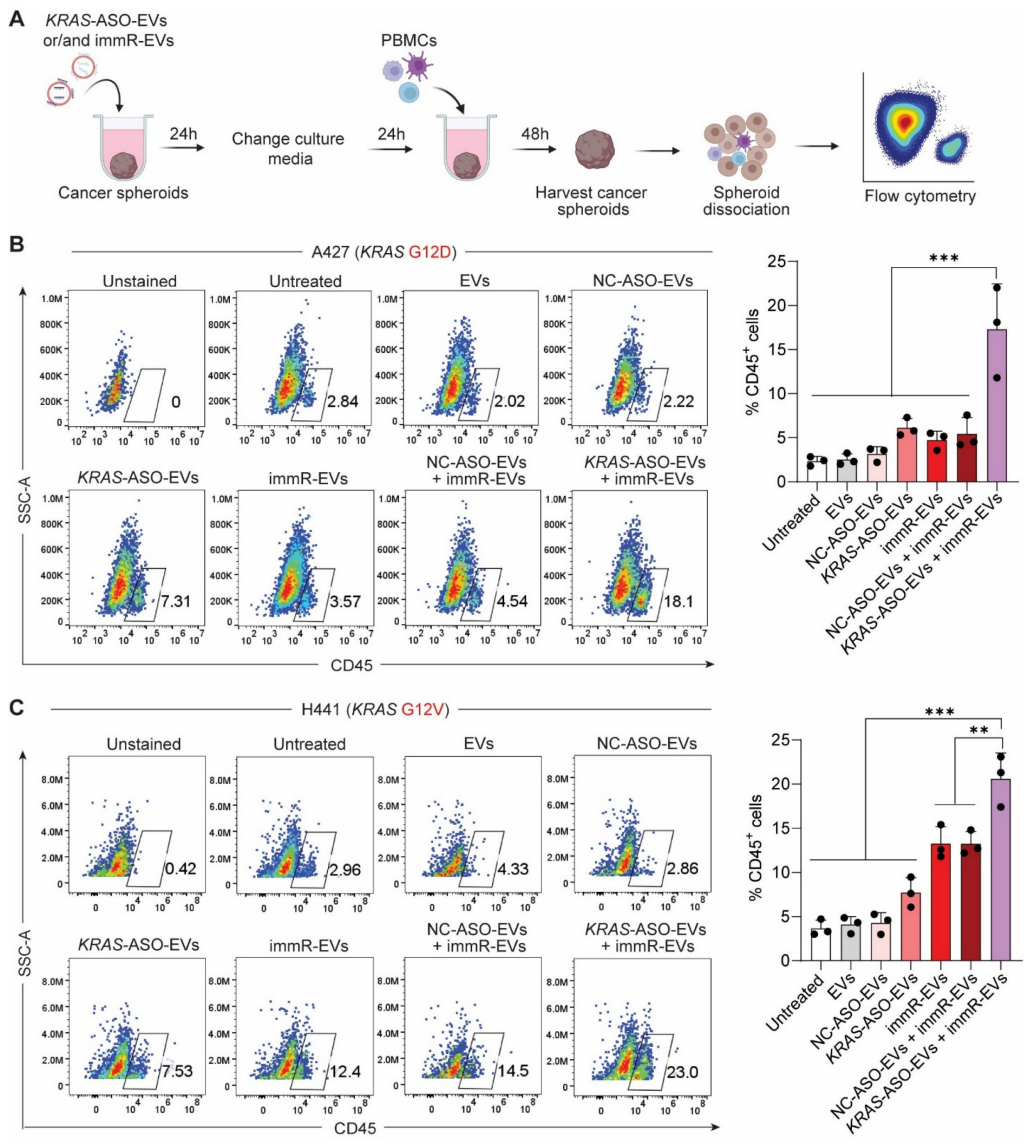
** Combination of *KRAS* ASO and immRNA delivered by RBCEVs promotes immune cell infiltration into KRAS-addicted cancer spheroids.** (**A**) Schematic for the evaluation of peripheral blood mononuclear cell (PBMC) infiltration into 3D cultures of A427 (*KRAS* G12D) and H441 (*KRAS* G12V) cells pre-treated with KRAS-ASO-EVs and/or immR-EVs, assessed by flow cytometry. (**B**-**C**) Flow cytometric analysis showing the percentage of CD45^+^ immune cells infiltrated into spheroids formed by (**B**) A427 and (**C**) H441 cells 48 h after the co-culture. The graphs present mean ± SEM. **P < 0.01, and ***P < 0.001, determined using One-Way ANOVA test.

Encouraged by the observation that the combination treatment potentiates the RIG-I activation in KRAS mutant-driven cancer cells, we investigated the infiltration of human peripheral blood mononuclear cells (PBMCs) into the cancer spheroids after the treatment (Figure 3A). Flow cytometric analysis (Figure 3B-C) showed that the combination treatment of *KRAS*-ASO-EVs and immR-EVs outperformed all other treatment groups in promoting the infiltration of CD45^+^ immune cells into cancer spheroids. Specifically, this combination induced the highest infiltration of monocytes and NK cells (Figure S8). These results suggest that co-treatment of *KRAS* mutant-targeting ASOs and RIG-I agonist RNA using the RBCEV platform may enhance immune cell infiltration and potentially promote a more immunologically active tumor microenvironment.

### Combining *KRAS* G12D ASO with immRNA in RBCEVs elicits a potent response against KRAS-driven tumors

Subsequently, we investigated the distribution of RBCEVs delivered with *Kras* ASO and immRNA in mice following intratracheal administration at various time points following intratracheal administration (Figure S9A). The result showed that the RBCEVs carrying ASO and immRNA predominantly localized in the lungs, with no significant changes in EV signals observed 24 h post-treatment (Figure S9B & S9C). We next examined the uptake of RBCEVs by *KRAS*-mutated tumor cells *in vivo*. We generated an orthotopic lung cancer model by injecting luciferase and mCherry-expressing A427 cells (Figure S10A & S10B) in the tail vein of NSGS mice. After 6 weeks, the tumor-bearing mice were followed up by intratracheal administration of Aco-490 dye-labeled EVs (Aco-490-EVs) (Figure S10C). At 3 h post-delivery, we assessed the uptake of RBCEVs by tumor cells using flow cytometry and confocal microscopy. Flow cytometric analysis demonstrated that RBCEVs were efficiently taken up by lung tumor cells (Figure S10D). Additionally, confocal imaging showed a significant co-localization of Aco-490-EVs with tumor cells (Figure S10E), highlighting the potential of RBCEVs as a drug carrier to lung cancer cells *in vivo*.

**Figure 4:**
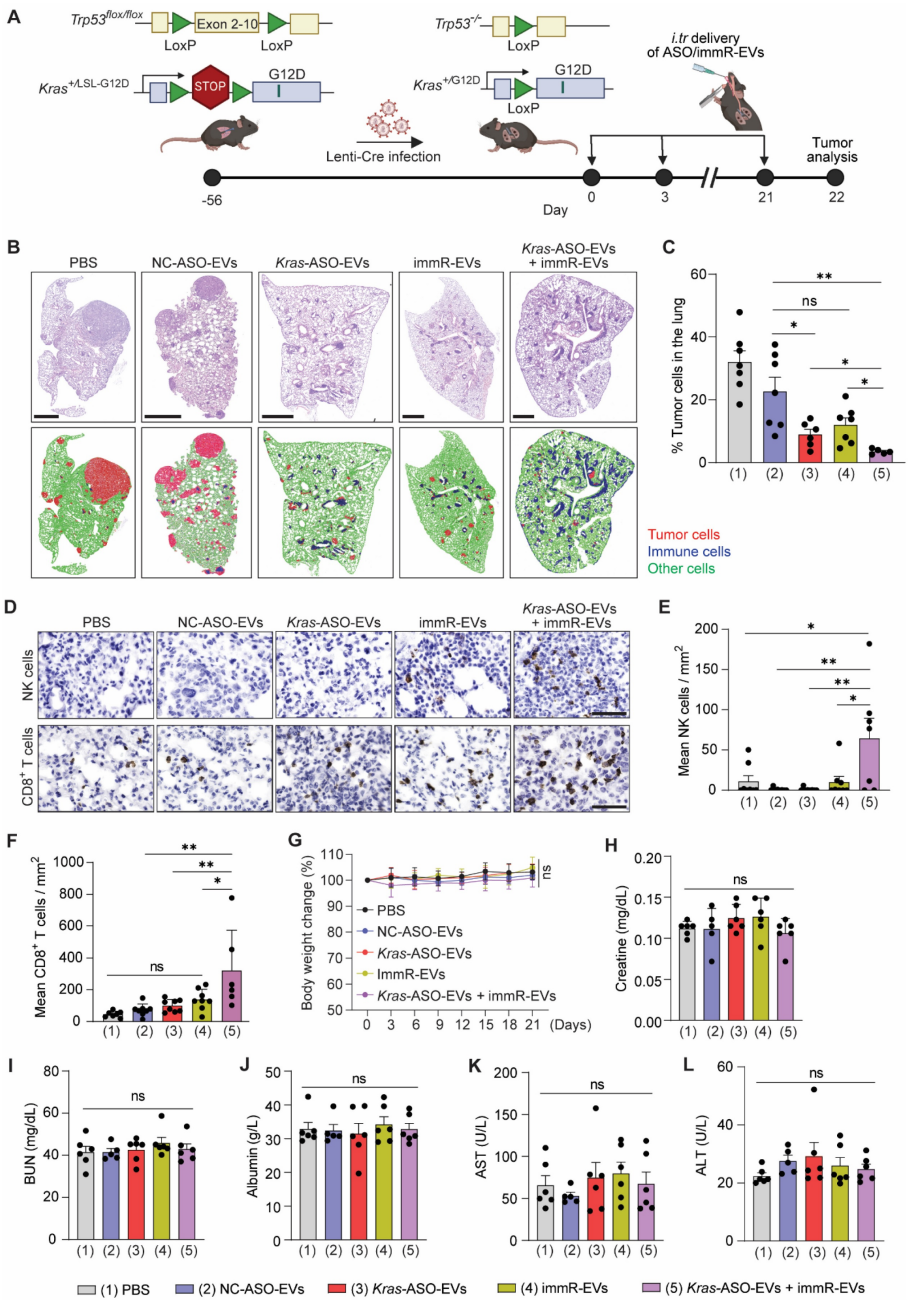
** Combination of *Kras* G12D ASO and immRNA in RBCEVs induces potent anti-cancer activity in a genetically engineered mouse model of NSCLC with *Kras* G12D mutation.** (**A**) Schematic for the tumor induction in the lung of *Kras^LSL-G12D^/+;p53^fl/fl^* (KP) mice and intratracheal administration schedule. 8 weeks (56 days) post-infection with Cre recombinase-expressing lentivirus (Lenti-Cre), the infected KP mice were intratracheally administered with (1) PBS or RBCEVs loaded with either (2) NC ASO (NC-ASO-EVs, 20 mg/kg RBCEVs), (3) *Kras* G12D ASO (*Kras*-ASO-EVs, 10 mg/kg RBCEVs), (4) immRNA (immR-EVs, 20 mg/kg RBCEVs), or (5) combined *Kras* G12D ASO and immRNA (*Kras*-ASO-EVs + immR-EVs, 20 mg/kg RBCEVs) every three days for three weeks (n = 6-7 mice/group). (**B**) Representative images of Haematoxylin and eosin (H&E) stained lung tissues from KP mice at the end of the study. Cell types were detected and analyzed using QuPath software. Red: tumor cells, blue: immune cells, green: other cell types. Scale bar: 1 mm. (**C**) Percentages of tumor cells in the lung of treated mice as described in (B) detected by QuPath software. (**D**) Representative immunohistochemistry (IHC) images of NK1.1 and CD8 protein expression (brown) in lung tumors of treated mice at the end of the study. (**E**-**F**) Densities of (**E**) NK cells and (**F**) CD8^+^ T cells within tumor areas quantified by IHC analysis as described in (D). (**G**) Change in body weight of mice receiving treatments as described in (A). (**H**-**L**) Serum concentrations of (**H**) Creatin, (**I**) blood urea nitrogen (BUN), (**J**) Albumin, (**K**) Aspartate-Aminotransferase (AST), and (**L**) Alanine Transaminase (ALT) in the treated mice as described in (A) at the end of the study. All bar graphs represent mean ± SEM. Ns - not significant, *P < 0.05, **P < 0.01, and ***P < 0.001 determined by One-Way ANOVA test.

To investigate the efficacy of the combination of *KRAS* ASO and immRNA in RBCEVs in an immunocompetent mouse model harboring a KRAS-mutated “cold” tumor, we obtained a genetically engineered mouse model (GEMM) with *Kras* G12D and p53 conditional mutations, known as *Kras^LSL-G12D/+^;p53^fl/fl^* (KP) mice from the Jackson Lab. We generated NSCLC in KP mice by infecting them with Cre recombinase-expressing lentivirus (Lenti-Cre) via intratracheal administration, leading to recombination and activation of the *Kras* mutant gene (Figure 4A & S11A) 34. We monitored and confirmed the formation of tumors in the lung of KP mice after Lenti-Cre infection by Haematoxylin and eosin (H&E) staining at different time points post-infection (Figure S11B). The therapeutic efficacy of the RBCEVs loaded with either NC ASO (NC-ASO-EVs), *Kras* G12D ASO (*Kras*-ASO-EVs), immRNA (immR-EVs), or combined *Kras* G12D ASO and immRNA (*Kras*-ASO-EVs + immR-EVs) was initially validated in this conditional NSCLC mouse model via intratracheal administration every three days (Figure 4A). We found that the treatment with *Kras*-ASO-EVs, immR-EVs, or combination of *Kras*-ASO-EVs and immR-EVs significantly dampened tumor growth as compared to the control treatment with PBS or NC-ASO-EVs, as indicated by the significantly reduced tumor cell density within the lung (Figure 4B-C). Notably, the *Kras* ASO + immRNA combination treatment exhibited the most superior anti-tumor effect compared to monotherapies. Intriguingly, immunohistochemistry (IHC) analysis revealed a significant increase in the infiltration of natural killer (NK) and CD8^+^ T cells (Figure 4D-F) in tumors of mice that received the *Kras* ASO + immRNA combination treatment, surpassing all other treated groups. Crucially, no significant changes were observed in all treated groups with regards to body weight (Figure 4G), histopathological morphology of major organs (Figure S12), and serum chemistry representing liver and kidney function, including Creatine (Figure 4H), blood urea nitrogen (BUN) (Figure 4I), Albumin (Figure 4J), Aspartate-Aminotransferase (AST) (Figure 4K), and Alanine Transaminase (ALT) (Figure 4L), underscoring the safety of the treatment.

**Figure 5:**
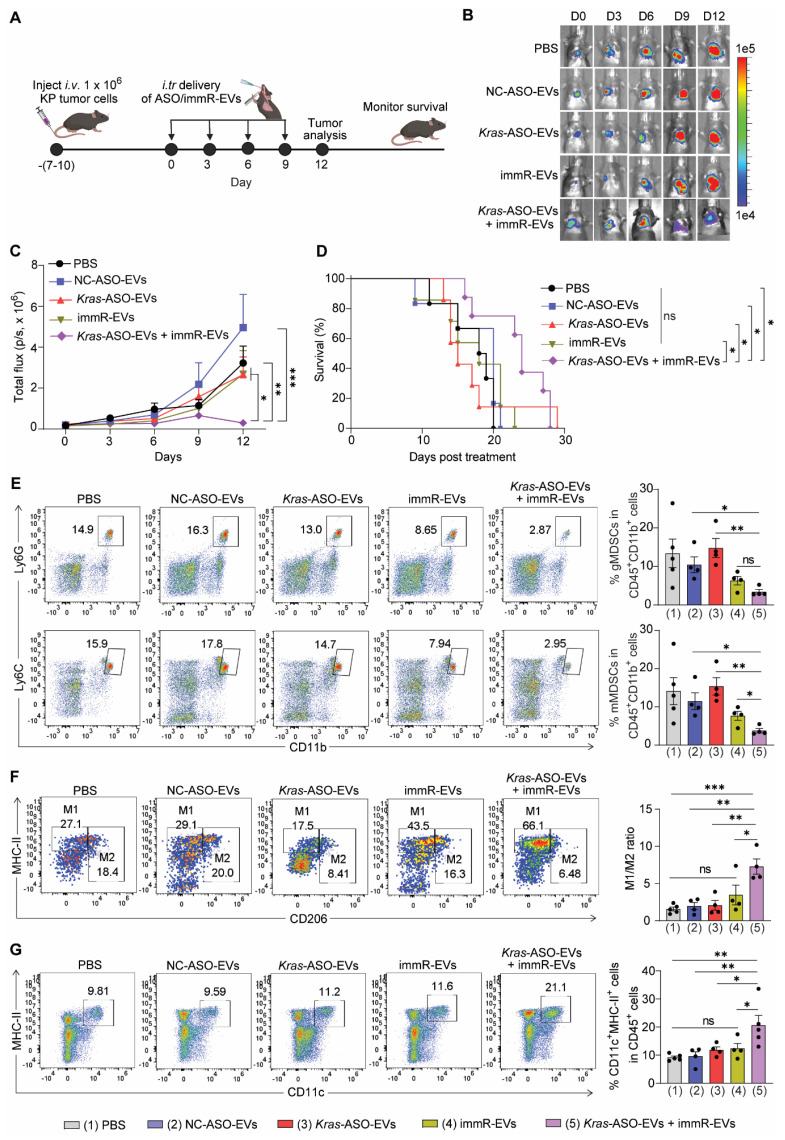
** Combination treatment of *Kras* ASO and immRNA in RBCEVs induces potent anti-cancer effect against aggressive cold tumors.** (**A**) Schematic for generating aggressive orthotopic lung tumor with mCherry-Luc-KP tumor cells in C57BL/6 mice and an intratracheal administration schedule. Mice with strong tumor signal at the lung were administrated with PBS or RBCEVs loaded with either NC ASO (NC-ASO-EVs, 20 mg/kg RBCEVs), *Kras* G12D ASO (*Kras*-ASO-EVs, 10 mg/kg RBCEVs), immRNA (immR-EVs, 10 mg/kg RBCEVs), or combined *Kras* G12D ASO and immRNA *(Kras*-ASO-EVs + immR-EVs, 20 mg/kg RBCEVs) every three days. (**B**) Representative bioluminescent images of orthotopic KP lung tumors expressing luciferase in treated xenograft mice over time. (**C**) Quantification of tumor progression in mice using the average bioluminescent signals following treatments over time (n = 4-5 mice/group). (**D**) Kaplan-Meier survival curves of treated mice as described in (A) (n = 6-8 mice/group). (**E**-**G**) Representative flow cytometry plot (left) and quantification (right) of (**E**) granulocytic and monocytic myeloid-derived suppressor cells (gMDSCs and mMDSCs, respectively) in CD45^+^CD11^+^ cells, (**F**) M1- and M2-like tumor-associated macrophages, and (**G**) populations of CD11c^+^MHC-II^+^ dendritic cells within the lung of treated mice at the end of study as described in (A) and (B). The graphs present the mean ± SEM. Ns - not significant, *P <0.05, **P < 0.01, ***P < 0.001 determined by two-way ANOVA (C), Gehan-Breslow-Wilcoxon test (D), or One-Way ANOVA test t-test (E-G).

Subsequently, we isolated EpCAM^+^CD45^-^ tumor cells (KP tumor cells) using fluorescence-activated cell sorting (FACS) from the lung of conditional genetic NSCLC KP mice (KP tumor cells) (Figure S11C-D) and transduced them with a plasmid expressing mCherry and luciferase to obtain mCherry and luciferase-expressing KP tumor cells (KP-mCherry-Luc tumor cells) (Figure S13). We next generated an aggressive orthotopic model of NSCLC with *Kras* G12D mutation by intravenously injecting KP-mCherry-Luc tumor cells into C57BL/6 mice. Following the development of a significant tumor burden in the lungs, we conducted the treatment in the same manner as described for the conditional genetic NSCLC mouse model (Figure 5A). It is noteworthy that in this aggressive model with a high tumor burden, *Kras*-ASO-EVs and immR-EVs failed to delay the tumor growth nor to prolong the survival rate of treated mice (Figure 5B-C). In contrast, the *Kras* ASO and immRNA combination treatment exhibited a potent anti-tumor effect, presenting the lowest tumor burden in the lungs (Figure 5C) and a significantly increased rate of survival among treated mice (Figure 5D).

Subsequent flow cytometry analysis of immune cells in the lungs of treated mice revealed that the *Kras* ASO and immRNA combination treatment led to a remarkable decrease in the number of immunosuppressive cells, including monocytic and granulocytic myeloid-derived suppressor cells (mMDSCs and gMDSCs, respectively) (Figure 5E), while increasing the ratio of M1/M2-like tumor-associated macrophages (Figure 5F) and number of DCs (Figure 5G) in the lung of treated mice. These findings indicate that the combination treatment demonstrates the potential to reverse the immunosuppressive TME in KRAS-mutated cancer to an immunologically responsive TME for effective cancer immunotherapy.

**Figure 6:**
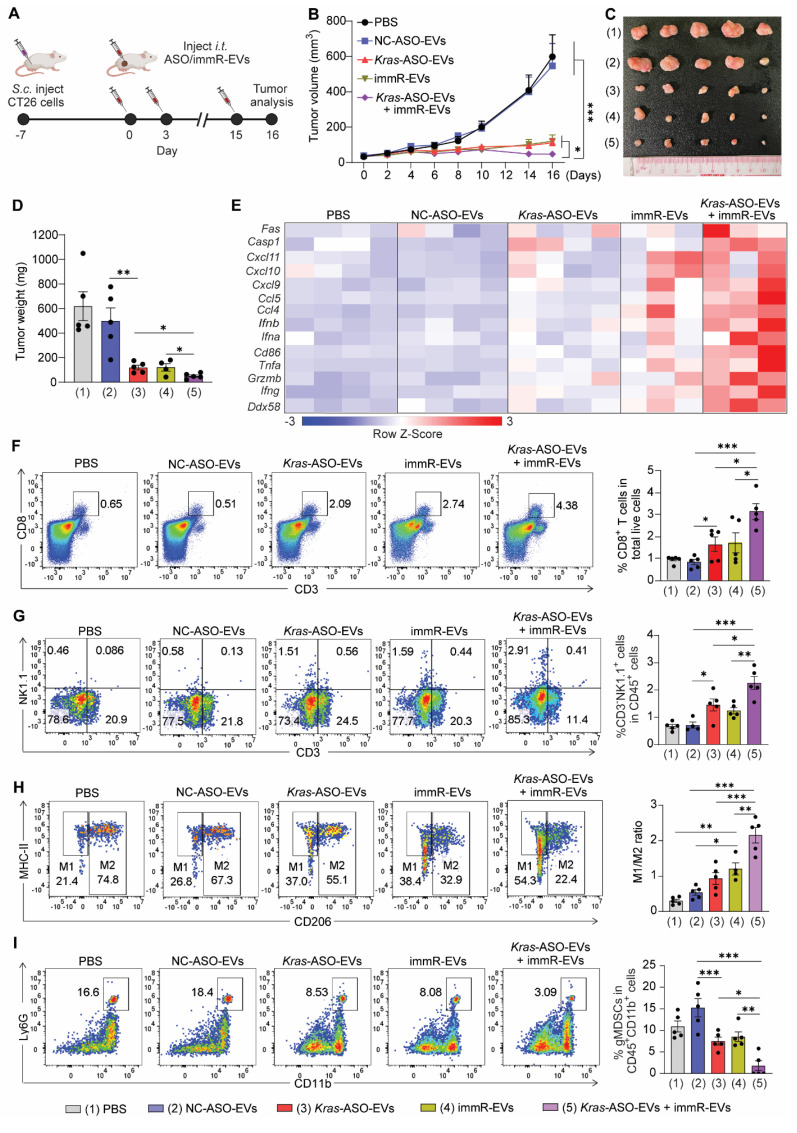
** Combination of *KRAS* ASO and immRNA shapes the immunogenic and tumoricidal tumor microenvironment of CT26 tumor**. (**A**) Intratumoral (*i.t.*) administration scheme for BALB/c mice with CT26 tumor. Mice with 50 mm^3^ subcutaneous tumors were intratumorally administered with PBS or RBCEVs loaded with either NC ASO (NC-ASO-EVs, 10 mg/kg RBCEVs), *Kras* G12D ASO (*Kras*-ASO-EVs, 5 mg/kg RBCEVs), immRNA (immR-EVs, 5 mg/kg RBCEVs), or combined *Kras* G12D ASO and immRNA *(Kras*-ASO-EVs + immR-EVs, 10 mg/kg RBCEVs) every three days, n = 5 mice/group. (**B**) Tumor volume of the CT26 tumor-bearing mice over time after the treatment. (**D**) Weight of tumor masses collected from treated mice at the end of the study. (**E**) Heatmap visualization of gene expression in tumors collected from treated mice at the end of the study using Gplots package in R software. (**F**-**I**) Representative flow cytometry histogram (left) and quantification (right) of (**F**) CD8^+^ T cells and (**G**) NK cells in total live tumor single cells, (**H**) M1- and M2- like tumor-associated macrophages, and (**I**) granulocytic myeloid-derived suppressor cells in intratumoral CD45^+^CD11^+^ cells. The graphs present the mean ± SEM. *P <0.05, **P < 0.01, ***P < 0.001 determined by two-way ANOVA (B) or One-Way ANOVA test (D, F-I).

We extended our investigation of the anticancer potency of the *Kras* ASO and immRNA combination treatment to a syngeneic mouse model of colorectal cancer harboring *Kras* G12D mutation. This tumor model was generated by subcutaneously (s.c) inoculating CT26 colorectal cancer cells into BALB/c mice followed by intratumoral injection with indicated EV formulations (Figure 6A). We found that the single treatment with *Kras*-ASO-EVs or immR-EVs significantly dampened the CT26 tumor growth as compared to PBS and NC-ASO-EVs (Figure 6B-D). Consistently, the combination of *Kras* G12D ASO and immRNA exhibited a significant tumor inhibition effect, indicated by reduced tumor volume (Figure 6B-C) and tumor weight (Figure 6D).

At the end of the treatment, we evaluated the gene expression profiles in the tumor tissues of treated mice. As expected, the combination treatment of *Kras* G12D ASO and immRNA induced an inflamed and tumoricidal environment with significant increases in the expression of RIG-I gene (*Ddx58*), genes associated with T cell and NK cell activation (*Ifng, Grzmb*), proinflammatory cytokines (*Tnfa*), DC activation (*Cd86*), and type I IFNs (*Ifna, Ifnb*), leukocyte chemokines (*Ccl4*), and apoptosis (*Casp1*) (Figure 6E & S14A-N). By Western blot analysis, we confirmed the KRAS inhibition as a result of KRAS ASO-loaded EV treatments (Figure S14O). Importantly, we observed that only the combination treatment induced the secretion of IFN-β within the treated tumor as detected by ELISA analysis (Figure S14P). Moreover, by flow cytometric analysis, we detected that this combination regimen induced the highest level of infiltration of CD8^+^ T cells (Figure 6F) and NK cells (Figure 6G), increased ratio of M1/M2-like tumor-associated macrophage (Figure 6H), and reduced gMDSC populations (Figure 6I) in the tumors of treated mice much more than all other treated groups.

### Combination of *Kras* ASO and immRNA induces immunogenic cell death effect in KRAS-mutated tumor models

**Figure 7:**
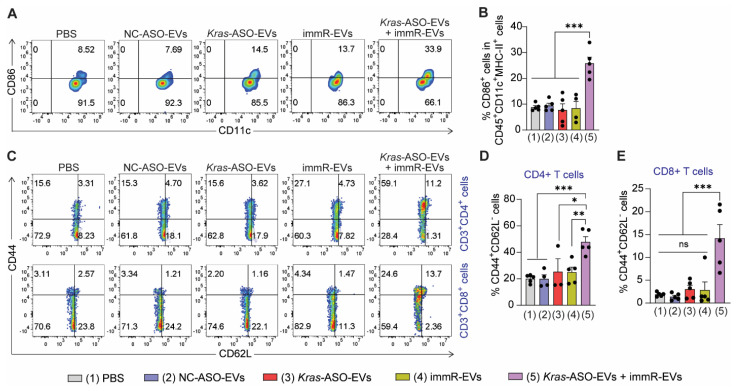
** Combination of *Kras* ASO and immRNA induces immunogenic cell death effect in a CT26 tumor model**. (**A**) Representative flow cytometry plot and (**B**) quantification of CD86 expression in dendritic cells at the tumor-draining lymph node of treated mice at the end of the study. (**C**) Representative flow cytometry plot and quantification of (**D**) effector memory CD4^+^ T cells (CD3^+^CD4^+^CD44^+^CD62L^-^) and effector memory CD8^+^ T cells (CD3^+^CD8^+^CD44^+^CD62L^-^) in the spleen of treated mice at the end of the study. The graphs present the mean ± SEM. *P <0.05, **P < 0.01, ***P < 0.001 determined by One-Way ANOVA test.

As the combination of *KRAS* ASO and immRNA in RBCEVs displayed a high ability to upregulate the expression of IFNs and induce tumor cell death in both *in vitro* and* in vivo* settings, we studied the induction of immunogenic cell death effect *in vivo* by quantifying the DC maturation markers (CD86) in tumor-draining lymph node (tdLNs) and the formation of effector memory T cells in the spleen. In the CT26 tumor model, the combination of *Kras* G12D ASO and immRNA led to enhanced DC maturation in the tdLN at a higher magnitude than the single treatments (Figure 7A-B). Given the importance of establishing the memory feature in cancer immunotherapy that provides long-term treatment benefits, we examined the percentage of effector memory CD4^+^ (CD3^+^CD4^+^CD44^+^CD62L^-^ cells) and CD8^+^ T cells (CD3^+^CD8^+^CD44^+^CD62L^-^ cells) in splenocytes of treated mice. As shown in Figure 7C-E, the *Kras* ASO and immRNA combination treatment resulted in significantly higher effector memory CD4^+^ and CD8^+^ T cells than all other treatments in the CT26 tumor model. In the aggressive “cold” KP tumors, this combination therapy induced higher levels of effector memory CD4^+^ and CD8^+^ T cells than the monotherapies of *Kras*-ASO-EVs or immR-EVs. Importantly, this combination treatment markedly outperformed NC-ASO-EVs in the generation of memory T cells (Figure S15). These results suggest the capability of the *KRAS* ASO and immRNA combination therapy delivered via RBCEVs to elicit a long-lasting immune response against the tumor.

## Discussion

Over the past decades, numerous therapeutic strategies have attempted to target *KRAS* mutants, including direct or indirect approaches encompassed downstream signaling pathways 35, epigenetic pathways 36, or the utilizing synthetic lethality approaches 37. Nevertheless, the majority of these approaches have proven suboptimal in efficacy and specificity. Although Sotorasib, a small molecule drug, has received FDA approval as an effective inhibitor of KRAS G12C, its application does not extend to other *KRAS* mutations, including other G12 mismatches. Moreover, a high proportion of patients who received Sotorasib experience disease relapse shortly after an initial response 38. Acquired resistance to this KRAS inhibitor eventually occurs in most patients, which is a persistent obstacle associated with targeted therapies 14. Therefore, there is a highly pressing clinical need to develop other effective KRAS inhibitors that can specifically target other KRAS mutants beyond G12C. Additionally, developing effective combination regimens is essential to overcome resistance mechanisms that emerge during treatment with KRAS mutant inhibitors. While immune checkpoint inhibitors (ICIs) have shown significant clinical benefits in many types of cancers, their efficacy in “immune cold” KRAS-addicted tumors has been disappointing even when combined with Sotorasib. Although Sotorasib could partially shift the TME to be more immunogenic, it is insufficient to achieve desired responses to ICIs 39.

Targeting KRAS mutants using small molecules is significantly challenging due to the lack of surface-binding pockets and the high affinity of the nucleotide-binding site in KRAS protein for GTP. Utilizing ASOs to target KRAS mutants at the RNA level may circumvent these limitations. ASOs offer numerous advantages compared to small molecules as they are less time-consuming and more cost-effective to design and synthesize 40. Here, we have successfully designed mutant-specific ASOs for selectively silencing mutations in the *KRAS* gene in various tumor cell types, while minimizing the cross-reaction with wild-type genes. Unlike previous ASO-based approaches that lacked specificity for KRAS mutations, our ASO design ensures selectively targeting mutant KRAS, thereby maximizing therapeutic efficacy while reducing off-target effects.

In a previous study 26, we reported a safety profile and potent anti-cancer effects of RBCEVs-delivered immRNA, mediated by the stimulation of RIG-I. Building upon these findings and clinical evidence that KRAS inhibitors are able to drive IFN-mediated antitumor immunity through reversing the repression of IRFs in KRAS-mutated cancers 7,39, we propose that the KRAS inhibitors can synergize with concomitant RIG-I activation to amplify the anti-tumor potency in KRAS-addicted cancers. This is particularly critical as resistance to KRAS inhibitors can further enhance the aggressive tumor immunosuppressive microenvironment, making the tumor even less responsive to immunotherapy 41. Our data demonstrated that the combination of *KRAS* ASO with RIG-I agonist significantly induced cell death across all tested KRAS-mutated tumor cell types *in vitro*. These findings also translated into robust suppression of tumor growth in immunocompetent mouse models of KRAS-driven colorectal and NSCLC. This study expands upon our previous work by introducing a novel combinatorial therapeutic strategy that simultaneously targets oncogenic KRAS while engaging the innate immune system via RIG-I activation. Of importance, this synergistic approach not only disrupts KRAS-driven oncogenesis but also amplifies immunostimulatory signaling, culminating in enhanced tumor suppression even in poorly immunogenic tumor models. We show that in an aggressive “cold” NSCLC model, only this combination treatment effectively delayed the tumor growth and increased the survival rate. This potent anti-cancer effect involves the induction of tumor cell death and the conversion of immunosuppressive tumors to immunogenic and tumoricidal microenvironments, accompanied by the augment of IFNs and pro-inflammatory cytokines, resulting in the enhanced recruitment of CD8^+^ T cells and NK cells, activation of DCs, and reduced populations of immunosuppressive cells (M_2_-like TAM and MDSCs). The finding that KRAS inhibition augments RIG-I-induced immune responses underscores an interplay between oncogene silencing and innate immune activation, highlighting the translational potential of this combination therapy for KRAS-mutated cancers resistant to conventional treatments. It is important to note that KRAS inhibition, when combined with RIG-I activation, can significantly elevate the formation of effector memory T cells in the CT26 tumor model, which exhibits a "hot tumor" profile characterized by highly infiltrated immune cells 42, Interestingly, even in the "cold" KP tumors, the *Kras* ASO and immRNA combination treatment partially increased the formation of effector memory T cells, suggesting that this approach may provide a durable anti-cancer effect. In addition, we speculate that subsequent treatment with other immunotherapeutic modalities, such as ICIs, following this combination treatment may further potentiate anti-cancer immune responses as tissue-resident memory T cells are capable of rapidly responding to ICIs 43.

While our study provides compelling evidence supporting the efficacy of *KRAS* ASO and RIG-I agonist combination therapy, further study is still required to translate this therapeutic prototype from bench to bedside. First, our current study is limited to cell lines and mouse cancer models. Further validation of the therapy in patient-derived xenografts and large animal models is important to confirm its translational potential. Second, the precise molecular mechanisms underlying the synergy between KRAS inhibition and RIG-I activation require further investigation to elucidate the downstream immune-modulatory pathways. Additionally, the potential for immune-related adverse events arising from prolonged RIG-I activation should be explored to ensure the safety of this therapeutic strategy.

Optimizing RBCEV-based delivery systems for *KRAS* ASO and immRNA to enhance systemic administration efficiency remains a critical area for further research. Moreover, exploring the potential for combining this regimen with immune checkpoint inhibitors or other immune-modulatory agents could further augment its therapeutic efficacy in resistant tumor settings. Expanding this strategy to target additional oncogenic drivers beyond KRAS may also unlock new opportunities for addressing cancers with high unmet medical needs.

By demonstrating a robust and durable anti-tumor effect in "cold" tumor models, this work presents a significant advancement over existing KRAS-targeted therapies, addressing both tumor cell-intrinsic and immune-mediated resistance mechanisms. Taken together, our findings underscore the promise of mutated KRAS inhibition with innate immune stimulation as a novel therapeutic paradigm for KRAS-driven cancers.

## Experimental Section

*RBCEV purification*: RBCEVs were purified as previously described 28. O-group human blood units were obtained from Innovative Research Inc. (USA), and EV purification was performed at Esco Aster (Singapore). Briefly, blood cells were separated from plasma by centrifugation, followed by a leukocyte removal using a leukodepletion filter (Nigale Biotechnology, China). EV release from RBCs was induced by adding calcium ionophore (Sigma Aldrich, USA) to the final concentration of 10 µM and incubating at 37 °C overnight in an incubator. EVs were collected from the RBC culture supernatant by sequential centrifugation at 600 × g for 20 min, 1600 × g for 15 min, and 3260 × g for 15 min at 4 °C to remove cells and debris. Any remaining debris was removed by filtering the supernatant through 0.45 μm-syringe filters. EVs were then concentrated by ultracentrifuging at 50,000 × g for 60 min at 4 °C using a TY70Ti rotor (Beckman Coulter, USA). To remove contaminated proteins, EVs were layered on top of 60% sucrose, and cushion centrifugation was carried out at 50,000 × g for 16 h at 4 °C using a SW41Ti swing rotor (Beckman Coulter, USA) without brake. The thin layer in the middle containing EVs was collected and washed again with PBS and concentrated using the TY70Ti rotor at 50,000 × g for 70 min at 4 °C. EVs were then resuspended in 4% Trehalose (Sigma Aldrich, USA) and stored at -80 °C for further uses.

*EV characterization*: Since haemoglobin (Hb) is the major content in RBCEVs, EV amounts were quantified by measuring the Hb quantity using Nanodrop (Thermo Fisher Scientific, USA). The particle concentration of RBCEVs was determined using a ZetaView® nanoparticle tracking analyzer (Particle Metrix, Germany). Size distribution and surface charge of EVs were determined by the dynamic light scattering (DLS) method using a Zetasizer Ultra instrument (Malvern Panalytical, UK). The morphology of RBCEVs and ASO-loaded RBCEVs was characterized by transmission electron microscopy (TEM). Briefly, RBCEVs were fixed with 2% paraformaldehyde, mounted on glow-discharged copper grids, and washed. Negative staining by incubating with 3% uranyl acetate was followed by washing and air-drying. Images of RBCEVs were captured using a Tecnai G2 transmission electron microscope at 100 kV (FEI/Philips, USA).

*EV stability:* To assess the stability of RBCEVs under different buffer conditions and temperatures, RBCEVs were incubated in various conditions, including 4% trehalose (storage buffer), PBS pH 7.4 (physiological pH), 50 mg/mL BSA in PBS (mimicking blood protein concentration), and PBS pH 6.0 (acidic condition of the tumor microenvironment) at either 4 °C or 37 °C. EV concentration, size, and surface charge were measured using nanoparticle tracking analysis and DLS method after 24, 48, and 72 h of storage in each condition.

*Synthesis of ASOs and immRNA*: ASOs targeting human *KRAS* G12D, *KRAS* G12V, and mouse *Kras* G12D, negative control ASO (NC ASO, 5'-CGACTATACGCGCAATATGG-3'), and Alexa Fluor 488-labeled NC ASO (AF488-ASO) were chemically modified with 2'-O-methoxyethyl (2'-MOE), phosphorothioate (PS), and locked nucleic acid (LNA) (Figure S2A) and synthesized by Integrated DNA Technologies (IDT, USA). Immunomodulatory RNAs (immRNAs) were synthesized as previously reported 26.

*ASO and immRNA loading onto RBCEVs*: ASO or immRNA was loaded onto RBCEVs using REG-1 reagent (Carmine Therapeutics, Singapore) following the manufacturer's protocol. Briefly, ASO or immRNA were incubated with REG-1 in Opti-MEM medium (Thermo Fisher Scientific, USA) at a final concentration of 10 µg/mL for ASO or immRNA and 70 µg/mL for REG-1, respectively, for 10 min at 25 ^o^C. Then, 50 µg EVs (1 mg/mL in Opti-MEM medium) were incubated with the mixture of REG-1 and ASO/immRNA at 37 °C for 30 min with gentle rotation. Finally, free REG-1, ASO, and immRNA were washed away by centrifuging at 21,000 x g for 30 min.

To determine the loading efficacy of ASO or immRNA onto EVs, 20 µg of loaded EVs were resuspended in 16 uL of 0.1% Triton-X (Sigma Aldrich, USA) in nuclease-free water and incubated at room temperature for 5 min. Thereafter, 4 µL of 100 mg/mL heparin sulfate (Sigma Aldrich, USA) was added to the lysed EVs, followed by incubation at 37 °C for 1 h with shaking. Subsequently, 4 µL of 6X DNA loading dye (New England Biolabs, USA) was added to the resulting mixture, followed by electrophoresis on a 2% agarose gel at 100 volts. Different concentrations of free ASOs ranging from 31.25 ng to 500 ng were included as standards. Agarose gels were imaged on the Gel Documentation system (Gel Doc XR+, Bio-Rad Laboratories, USA), and the band densitometry was determined using ImageJ software.

To assess the stability of immRNA and ASO after loading onto RBCEVs, the loaded RBCEVs were stored in EV storage buffer (PBS supplemented with 4% trehalose) for up to 96 h. The percentage of immRNA and ASO remaining on 50 µg of loaded EVs was measured immediately after loading and at 24 h intervals by lysing the loaded RBCEVs and analyzing them on an agarose gel using the previously described protocol.

*ASO and immRNA loading into lipid nanoparticles (LNPs)*: ASO or immRNA-loaded LNPs were prepared using the dilution method. Briefly, 10 µg of ASO or immRNA in 500 µL of HEPES buffer (10 mM, pH 4.0) was added to 500 μL of ethanol containing 185 nmole of 1,2-dioleoyl-3-dimethylammonium-propane (DODAP, Avanti Polar Lipids, USA), 500 nmole of 1,2-dioleoyl-sn-glycero-3-phosphoethanolamine (DOPE, Avanti Polar Lipids, USA), 300 nmole of Cholesterol (Sigma Aldrich, USA), and 15 nmole of methoxypolyethyleneglycol 1,2 distearoyl-sn-glycero-3-phospho-ethanolamine (DSPE-mPEG, Nanocs, USA) under vortexing. Then, 4.5 ml of HEPES (10 mM, pH 4.0) was added to the solution to dilute the concentration of ethanol. The resulting solutions were centrifuged (4000 x g, 30 min, 20 °C) using Amicon Ultra 100K tube. The LNPs were next buffer-exchanged and concentrated by adding 4.0 ml of PBS pH 7.4 to the tubes and centrifuged again at 4000 x g, 30 min, 20 °C.

*Cell culture*: A427, H441, A549, AsPC1, CT26, and HEK-293T cells were obtained from the American Type Culture Collection (ATCC). A427, H441, AsPC-1, and CT26 cells were maintained in RPMI 1640 media (Thermo Fisher Scientific, USA). A549 and HEK293T cells were maintained in DMEM media (Gibco). All media were supplemented with 10% fetal bovine serum (FBS, Thermo Fisher Scientific, USA), 1% Penicillin/Streptomycin (Thermo Fisher Scientific, USA), and 1X Plasmocin (InvivoGen, USA).

Murine KP tumor cells were derived from lung tumors of Kras^+/G12D^;Trp53^-/-^ mice. Briefly, mouse lung tissues were cut into small pieces and digested with collagenase IV (Thermo Fisher Scientific, USA) for 20 min at 37 °C with gentle rotation. The cell suspension was then passed through a 70-µm cell strainer and centrifuged at 500 × g for 5 min. RBCs were lysed with ACK lysis buffer (Thermo Fisher Scientific, USA) for 10 min at room temperature, and the remaining cells were washed with FACS buffer (2% FBS, 2 mM EDTA in PBS). The collected cells were subsequently stained with anti-mouse EPCAM antibody (#ab71916, Abcam, USA) and anti-mouse CD45 antibody (PE-Cy7 conjugated, #147704, Biolegend, USA) for 30 min on ice. The stained cells were washed with FACS buffer and stained with anti-rabbit AF488 secondary antibody (#711-547-003, Jackson ImmunoResearch Laboratories, USA). The EPCAM^+^CD45^-^ cells were sorted out on an Aria II sorter (BD Biosciences, USA) and cultured in RPMI medium supplemented with 10% FBS. Recombined *Kras* and *Trp53* genes were validated by PCR genotyping using the primer sets listed in Table S1.

A human patient-derived pancreatic organoid line (PDO67) was kindly provided by Prof. Glenn Bonney (Cancer Science Institute of Singapore, National University of Singapore). The organoids, prepared in 95% Matrigel (Corning, USA), were cultured in advanced DMEM/F12 medium (Stemcell Technologies, USA).

All cells were cultured in the humidified chamber at 37 °C with 5% CO_2_.

*Toxicity of native RBCEVs on A427 cells:* To determine the half-maximal inhibitory concentration (IC50) of native RBCEVs on A427 cells, 1 × 10^4^ cells were seeded per well in a 96-well plate 16 h before treatment with different batches of RBCEVs at concentrations ranging from 25 to 1000 µg/mL. After 48 h, relative A427 cell counts were assessed using the Cell Counting Kit-8 (CCK-8) assay (MedChemExpress, USA) with a microplate reader (Tecan, Switzerland). Untreated cells served as controls.

*Toxicity of native RBCEVs on normal lung cells:* Human bronchial epithelial cells (HBECs) were seeded at a density of 1 × 10^5^ cells per well in 24-well plate 16 h prior to treatment. The seeded cells were treated with either unloaded RBCEVs (EVs), 10 mg/kg RBCEVs loaded with NC ASO (NC-ASO-EVs), or a combination of *KRAS* G12D ASO and immRNA (*KRAS*-ASO-EVs + immR-EVs) for 24 h. Flow cytometric analysis of Annexin V/PI staining was performed 24 h post-treatment to determine the proportion of live (Annexin-, PI-), early apoptotic (Annexin+, PI-), necrotic (Annexin-, PI+), and late apoptotic (Annexin+, PI+) cells.

*Sanger sequencing*: Oncogenic mutations in A427 (*KRAS* G12D) and H441 (*KRAS* G12V) cell lines were validated using Sanger sequencing. Genomic DNAs were first extracted from each cell line. The amplicons of exon 2, where mutations are located, were amplified by PCR using the same primer pair designed for Sanger sequencing (Table S2). Subsequently, the PCR-produced amplicons and primers were then sent to Macrogen Singapore for sequencing. Data provided was then analyzed and exported by GeneStudio software (version v.1.41).

*Lentivirus production*: All production steps of 3^rd^ generation of lentiviruses followed the NUS Laboratory Materials Management System (LMMS) guidelines. Packaging plasmids (pMDLg/pRRE and pRSV-REV) and envelop plasmid (VSV-G) were purchased from Invitrogen (USA). Plasmids encoding the Cre recombination enzyme (Puro-Cre, #17408) and mCherry-Luc-Blast (#161789) were obtained from Addgene. Plasmid encoding mCherry-Fluc-Puro was kindly provided by Prof. Andrew Kung, University of Columbia.

For lentivirus production, 5.0 x 10^6^ HEK-293T cells were seeded on 10-cm plates coated with Poly-D-Lysine (Gibco, USA) for 16 h before transfection. pMDLg/pRRE (3.25 µg), pRSV-REV (1.5 µg), VSV-G (1.5 µg), and the expression plasmid (5 µg) were co-delivered into HEK-293T cells using PEI_max_ transfection reagent (Polysciences, USA) in DMEM supplemented with 1% FBS. The media was replaced after 24 h with DMEM supplemented with 10% FBS and kept for 40 h before virus collection. Then, cells and debris were pelleted and removed by spinning at 1,000 x g for 10 min at 4 °C. The supernatant containing viruses was then passed through a 0.45 μM filter (Merck Millipore, USA) and concentrated using Amicon tubes (10,000 MWCO, Merck Millipore) at 2,400 x g for 1 h at 4 °C. Viral stocks were aliquoted into a small volume of 100 µL, snap-frozen in liquid nitrogen, and stored at -80 °C.

*mCherry and luciferase-expressing cell line generation*: pLenti-ARE-HSVTK-luc plasmid (Plasmid #161789, Addgene) was used to generate mCherry and luciferase-expression KP tumor cells (KP-mCherry-Luc cells). Briefly, 1.0 x 10^5^ freshly trypsinized KP cells were transduced with 3 x 10^4^ mCherry-Luc lentiviral particles in a total of 300 µL of RPMI medium supplemented with 10% FBS and 8 µg/mL of Polybrene (Sigma Aldrich, USA). The medium was refreshed 24 h post-infection. After three days, Blasticidin (InvivoGen, USA) was added to the cell culture medium at the final concentration of 7 µg/mL for selecting the transduced cells. The medium containing Blasticidin was replaced daily until the cell number reached at least 2-3 million for sorting. Five percent of the highest mCherry-expressing cells were sorted out using an Aria II sorter (BD Biosciences, USA).

The pLV-Fluc-mCherry-TM-PuroR plasmid was used for generating A427-mCherry-Luc cells, with puromycin selection, following the same procedure.

*In vitro uptake assay*: To analyze the uptake of RBCEVs by H441 and A427 cells, AF488-ASOs were loaded onto EVs following the protocol described above. 50,000 cells were seeded in each well of a 12-well plate and incubated for 16 h prior to the treatment. AF488-ASO-loaded EVs were then added to cells at different concentrations. After 2 or 4 h, cells were washed twice with FACS buffer, and AF488 ASO signal was detected by Flow Cytometry (CytoFlex, Beckman Coulter, USA).

*Western blot*: Cells or EVs were lysed in RIPA buffer (Thermo Fisher Scientific, USA) containing protease inhibitor (Roche, Switzerland) for 20 min on ice, followed by centrifugation at 20,000 x g for 20 min at 4 °C to collect supernatants. Protein concentration was determined using SMART™ BCA Protein Assay Kit (iNtRON Biotechnology, USA). Next, 30-50 µg of protein lysate was loaded onto Acrylamide gel (BioRad Laboratories, USA) and separated by size in 10% polyacrylamide gels. The proteins were subsequently transferred onto a methanol-activated polyvinylidene difluoride (PVDF) membrane (Merck Millipore, USA), followed by blocking with 5% skim milk for 30 min at room temperature. The membranes were then incubated overnight at 4 °C with primary antibodies, including anti-human BAND 3 (sc-133190, Santa Cruz, 1:1000), anti-human STOMATIN (sc-376869, Santa Cruz 1:1000), anti-human ALIX (sc-53538, Santa Cruz , 1:500), anti-human TSG101 (sc-7964, Santa Cruz, 1:500), anti-human HBA (sc-21005, Santa Cruz, 1:1000), anti-human GPA (#306602, Biolegend, 1:500), anti-human β-actin (HRP-60008, Proteintech, 1:5000), anti-human CANX (sc-23954, Santa Cruz, 1:500), and anti-human GAPDH (HRP-60004, Proteintech, 1:5000), anti-human KRAS (WH0003845M1-100UG, Sigma Aldrich, 1:1000), anti-mouse KRAS (sc-30, Santa Cruz, 1:1000). After washing with Tris Buffer Saline supplemented with 1% Tween-20 (TBST), membranes were incubated with HRP-conjugated secondary antibodies (VectorLabs, USA) for 2 h at room temperature. Finally, the stained membranes were washed with TBST and incubated with WesternBright ECL chemiluminescent HRP substrate (Advansta, USA) for detecting chemiluminescent signal using an XR+ Gel Documentation System (Bio-Rad Laboratories, USA).

*Cell viability and apoptosis assay*: Cells were seeded at a density of 1.0 x 10^4^ cells per well in a 96-well plate and incubated overnight prior to incubation with RBCEVs (25 µg/mL) or RBCEVs loaded with NC ASO (NC-ASO-EVs, 25 µg/mL RBCEVs), *KRAS* ASO (*KRAS*-ASO-EVs, 25 µg/mL RBCEVs), immRNA (immR-EVs, 25 µg/mL RBCEVs), combined NC ASO and immRNA (NC-ASO-EVs + immR-EVs, 50 µg/mL RBCEVs), or combined *KRAS* ASO and immRNA (*KRAS*-ASO-EVs + immR-EVs, 50 µg/mL RBCEVs). Cell viability was determined after 48 h by Cell Counting Kit-8 (CCK-8) assay (MedChemExpress, USA) in a microplate reader (Tecan, Switzerland).

Apoptosis of cancer cells after treatment was characterized by Annexin V (Biolegend, USA) and SYTOX Blue (Thermo Fisher Scientific, USA) staining. Briefly, cancer cells were seeded in 12-well plates at a density of 1.0 x 10^5^ cells per well prior to incubation with different RBCEV formulations as described above. After 24 h, cells were washed with PBS, harvested, and stained with Annexin V and SYTOX Blue following the supplier's instructions. The apoptotic cells were then detected by Flow Cytometry (CytoFlex, Beckman Coulter, USA).

*In vitro evaluation of RIG-I pathway activation by quantitative RT-PCR*: Cells were transfected with different EV formulations as described in the apoptosis assay. After 48 h, treated cells were washed, harvested, and lysed in Trizol reagent (Invitrogen, USA) for total RNA extraction. The extracted RNA concentration was determined by Nanodrop spectrophotometer (Thermo Fisher Scientific, USA). Then, 500 ng of RNA from each sample was conversed into cDNA using the high-capacity cDNA reverse transcription kit (Thermo Fisher Scientific, USA). The gene expression was quantified by quantitative real-time PCR (qRT-PCR) using SsoAdvanced Universal SYBR (Bio-Rad Laboratories, USA). *GAPDH* expression was included as an internal control for normalization among samples. All qRT-PCR reactions were conducted on a QuantStudio 6 Flex Real-Time PCR System (Life Technologies, USA) using the primers listed in Table S3.

*Assessment of PBMC infiltration into cancer spheroids*: Either A427 or H441 cells were seeded in ultra-low-attachment U-bottom 96-well plates at a density of 5,000 cells per well in 200 µL of RPMI medium and cultured in an incubator overnight. Thereafter, cell aggregation was initiated by centrifuging the plate for 5 min at 300 × g, 25 °C, followed by further incubation at 37 °C for two days to allow cells to grow and form more compact aggregates. Then, 100 µL of culture medium was replaced with 100 µL of 10% (v/v) Matrigel (Corning, USA), prepared in ice-cold RPMI (Thermo Fisher Scientific, USA). The plates were centrifuged for 5 min at 300 × g, 25 °C, and grown for two more days before the treatment with different RBCEV formulations as previously described. 24 h post-treatment, the culture media was removed and replaced with 100 µL of culture medium containing 5% (v/v) matrigel, and the cancer spheroids were cultured for another 24 h. After that, 50 μl of human PBMCs at a density of 5.0 x 10^6^ cells/mL was added into each well containing treated spheroids to achieve a final concentration of 1.0 x 10^6^ PBMCs per mL. After 48 h of co-culture, spheroids were first washed thrice with PBS to remove non-infiltrated PBMCs. Then, 200 μL of pre-warmed 1X collagenase in RPMI was added to each well and incubated at 37 °C for 10 min. Spheroids were next gently collected into tubes and centrifuged at 500 x g for 4 min. The supernatants were removed, and spheroids were washed with cold PBS. Single cells were then obtained by treating spheroids with TrypLE (Thermo Fisher Scientific, USA) for 20 min at 37 °C and washed twice with PBS. Single cells were then incubated with Human Fc Blocker (Human TruStain FcX, Biolegend, USA), followed by staining with AF488-conjugated anti-human CD45 antibody (Jotbody, Hong Kong). The stained single cells were then analyzed in a flow cytometer (CytoFlex, Beckman Coulter, USA).

*Mouse care and experimentation*: All animals were maintained at the animal facilities of the National University of Singapore and treated in accordance with an approved protocol (protocol number: R19-1195) granted by the Institutional Animal Care and Use Committee (IACUC). NSG-SGM3 (NSGS) mice and B6.129-*Kras^tm4Tyj+/-^*, *Trp53^tm1Brn+/-^* mice (heterozygous for the *Kras^tm4Tyj^* and homozygous for the *Trp53^tm1Brn^*) were purchased from the Jackson Laboratory (USA). BALB/C and C57BL/6 mice were purchased from InVivos (Singapore).

*In vivo biodistribution study*: RBCEVs were incubated with Aco-490 membrane dye (Acoerela, Singapore) in PBS at a final concentration of RBCEVs and Aco-490 was 50 µg/µL and 2 µM, respectively, for 1 h at 25 ^o^C. Then, the stained EVs were washed twice with PBS at 21000 x g for 20 min to remove unbound dyes.

An A427 lung orthotopic cancer model was generated by injecting intravenously (i.v) 1.0 x 10^6^ A427-mCherry-Luc lung cancer cells into 6-8-week-old NSGS mice. When the tumor was detected in the lung, the tumor-bearing mice were then intratracheally treated with Aco-490-labeled RBCEVs at a dose of 10 mg/kg RBCEVs. After 3 h, the mice were euthanized and lungs were harvested for the analysis of EV uptake by tumor cells using flow cytometry and a confocal microscope. For flow cytometric analysis, single lung cells were prepared by dissociating the lung tissues in a gentleMACS dissociator (Miltenyi, Germany) and incubating in collagenase IV (Life Technologies, USA) for 40 min at 37 °C. The cells were then filtered through a 70 μm cell strainer, and RBCs were lysed using ACK lysis buffer. The obtained single cells were then washed with FACS buffer and analyzed using CytoFlex equipment (Beckman Coulter, USA).

To evaluate the pharmacokinetic profile of loaded RBCEVs after intratracheal administration, a combination of Kras-ASO-loaded EVs and immRNA-loaded EVs were labeled with Acoerela 800 (Aco800) dye and delivered to mice bearing KP-mCherry orthotopic tumors at a dose of 20 mg/kg RBCEVs. At each time point (15 min, 30 min, 1 h, 4 h, and 24 h), four mice were sacrificed, and blood, along with major organs (lungs, spleen, liver, kidneys, heart, and gastrointestinal tract), were collected. The presence of labeled RBCEVs was assessed using the IVIS system by measuring Aco800 fluorescent signals in the collected samples.

*Evaluation of anti-cancer effect in a conditional genetic model of NSCLC*: B6.129-*Kras^tm4Tyj+/-^*, *Trp53^tm1Brn+/-^* mice were bred, and the 6-week-old mice heterozygous for the LoxP-STOP-LoxP-*Kras*G12D (LSL-*Kras*G12D) were intratracheally administered with approximately 10,000 Cre-encoding lentiviral particles. 8 weeks post-infection, the mice were equally allocated into five treatment groups, including PBS, NC-ASO-EVs (20 mg/kg RBCEVs), *KRAS*-ASO-EVs (10 mg/kg RBCEVs), immR-EVs (10 mg/kg RBCEVs), and combination of *KRAS*-ASO-EVs (10 mg/kg RBCEVs) and immR-EVs (10 mg/kg RBCEVs). The treatments were administered via the intratracheal route at a 3-day interval for 3 weeks. At the end of the study, mice were humanely euthanized. Blood serum, lung tissue, and other major organs were harvested for analysis. Tumor lesions in the lung and morphological alterations in other organs were analyzed by H&E staining. The intratumoral level of NK cells and CD8 T cells were examined by immunohistochemical (IHC) staining. The systemic toxicity of treatments was determined through serum chemistry analysis.

*Evaluation of anti-cancer effect in orthotopic syngeneic lung cancer model*: 1.0 x 10^6^ KP-mCherry-Luc tumor cells in 200 µL of serum-free RPMI media were intravenously injected into 6-8-week-old female C57BL/6 mice. The tumor development in the lungs of injected mice was monitored using an IVIS imaging instrument (PerkinElmer, USA). Once a high tumor burden was detected in the lungs, the mice were divided into groups and treated as described in the treatment protocol for the conditional genetic model.

*Treatment of CT26 subcutaneous tumors*: 5.0 x 10^5^ CT26 cells in 100 µL of serum-free RPMI media were injected subcutaneously into the flank of 6-week-old female BALB/C mice. On reaching sizes of approximately 50 mm^3^, tumors were treated intratumorally with 20 μL of PBS containing NC-ASO-EVs (5 mg/kg RBCEVs), *Kras*-ASO-EVs (2.5 mg/kg RBCEVs), immR-EVs (2.5 mg/kg RBCEVs), and combination of *Kras*-ASO-EVs (2.5 mg/kg RBCEVs) and immR-EVs (2.5 mg/kg RBCEVs) for every three days. The tumor size was measured every two days using a digital caliper. The tumor volume was calculated using the formula: Volume = ½ x length x width^2^. At the end of the study, mice were euthanized to collect tumors, inguinal lymph nodes, and spleens for analysis. The representative plots of flow cytometry analysis of immune cells from treated tumors are shown in Figure S17-19.

*Flow cytometric analysis of lung tissues in the orthotopic syngeneic lung cancer model*: Single cells were dissociated from the lungs of the treated mice with orthotopic KP lung tumors as previously described. Then, the obtained cells were incubated with anti-mouse CD16/32 antibodies (Biolegend, USA) for 10 min at room temperature prior to incubating with several panels of fluorophores-conjugated antibodies (Table S4). Dead cells were identified using SYTOX Blue Dead Cell Stain (Thermo Fisher Scientific, USA). Cells were washed twice, suspended in FACS buffer, and analyzed on CytoFlex flow cytometer (Beckman Coulter, USA). The representative plot is shown in Figure S16.

*Hematoxylin and Eosin (H&E) staining*: Whole tissues were fixed in 10% formalin (Thermo Fisher Scientific, USA) for 24 h. The tissues were then auto-processed overnight in a tissue processor (Leica TP 1020, Germany) before being embedded in paraffin and sectioned with a thickness of 4 µm using a microtome (Leica RM2135, Leica Biosystems, Germany). Sections were deparaffinized in Histoclear solutions, rehydrated, and stained with Hematoxylin for 1 minute. After washing with water, sections were then stained with Eosin for 30 seconds, dehydrated in increasing concentrations of ethanol, and covered with mounting media. The H&E staining slides were imaged using an EVOS M7000 Imaging System (Thermo Fisher Scientific, USA). QuPath software (version 0.4.3) was used for the whole slide image analysis 44.

*Immunohistochemistry (IHC) staining*: FFPE sections were dewaxed and rehydrated, followed by antigen retrieval in Sodium Citrate (pH 6, Thermo Fisher Scientific, USA) or EDTA (pH 9, Sigma Aldrich, USA) buffers at boiling temperature for 20 min. After cooling to room temperature, the sections were blocked with 3% hydrogen peroxide for 10 min, followed by 5% goat serum (CST) for 1 h. Primary antibodies detecting mouse NKP46 (#ab233558, Abcam, 1:500), CD8 (#ab217344, Abcam, 1:500) were prepared in SignalStain® Antibody Diluent (#8112, CST). The sections were then incubated overnight at 4 °C in a humidity chamber. After aspirating the primary antibody, sections were washed twice with 1X Tris-Buffered Saline and incubated with anti-mouse HRP-conjugated secondary antibody (VectorLabs, 1:500) at room temperature for 60 min. After washing away the unbound secondary antibody, DAB chromogen solution (Cell Signaling Technology, USA) was added and incubated for 1-10 min to allow color development. Finally, the sections were counterstained with Hematoxylin to visualize cell nuclei, dehydrated, and covered with mounting media before imaging with the EVOS M7000 Imaging System (Thermo Fisher Scientific, USA).

*Serum chemistry analysis*: At the end of the study, blood was drawn from treated mice by cardiac puncture. The blood samples were left at room temperature for 30 min to allow clotting, and sera were collected by centrifuging the samples at 2,000 x g for 10 min at 4 °C. Serum blood chemistry was analyzed by the Veterinary Diagnostic Laboratory, NUS Comparative Medicine.

*Statistical analysis*: All quantitative data were presented on the graphs as mean ± standard error of the mean (SEM). Statistical analysis was performed on GraphPad Prism 9 (GraphPad Software, CA). For comparison between two groups, a two-tail Student's t-test (normal sample distribution) or Mann-Whitney test (data do not follow a normal distribution) was used, while one- or two-way ANOVA with Post Hoc Bonferroni correction was used when comparing more than 2 groups. A P-value below 0.05 from at least 3 biological replication treatments was considered statistically significant.

## Supplementary Material

Supplementary figures and tables.

## Abbreviations

EVs: extracellular vesicles
RBCEVs: red blood cell-derived extracellular vesicles
ASOs: antisense oligonucleotides
immRNA: immunomodulatory RNAs
KRAS: Kirsten rat sarcoma virus
RIG-I: retinoic acid-inducible gene-I
IFNs: interferons
NSCLC: non-small cell lung cancer
PBS: phosphate buffered saline
NC: negative control
IVIS: in vivo imaging system
I.V.: intravenous
S.C.: subcutaneous
TME: tumor microenvironment
STOM: Stomatin
CANX: Calnexin
HBA: Hemoglobin A
PDI: polydispersity index
NK: natural killer
TAM: tumor-associated macrophage
MDSC: myeloid-derived suppressor cells
MOE: 2'-O-methoxyethyl
PS: phosphorothioate
LNA: locked nucleic acid
PEI: Polyethylenimine
PDVF: polyvinylidene difluoride
IHC: immunohistochemistry
FFPE: formalin-fixed paraffin-embedded
HRP: horseradish peroxidase
TEM: transmission electron microscope

## References

[1] Simanshu DK, Nissley DV, McCormick F (2017). RAS Proteins and Their Regulators in Human Disease. Cell.

[2] Buscail L, Bournet B, Cordelier P (2020). Role of oncogenic KRAS in the diagnosis, prognosis and treatment of pancreatic cancer. Nat Rev Gastroenterol Hepatol.

[3] Jonckheere N, Vasseur R, Van Seuningen I (2017). The cornerstone K-RAS mutation in pancreatic adenocarcinoma: From cell signaling network, target genes, biological processes to therapeutic targeting. Crit Rev Oncol Hematol.

[4] Delpu Y, Hanoun N, Lulka H (2011). Genetic and Epigenetic Alterations in Pancreatic Carcinogenesis. Curr Genomics.

[5] Bournet B, Muscari F, Buscail C (2016). KRASG12D Mutation Subtype Is A Prognostic Factor for Advanced Pancreatic Adenocarcinoma. Clin Transl Gastroenterol.

[6] Bournet B, Buscail C, Muscari F, Cordelier P, Buscail L (2016). Targeting KRAS for diagnosis, prognosis, and treatment of pancreatic cancer: Hopes and realities. Eur J Cancer.

[7] Brägelmann J, Lorenz C, Borchmann S (2021). MAPK-pathway inhibition mediates inflammatory reprogramming and sensitizes tumors to targeted activation of innate immunity sensor RIG-I. Nat Commun.

[8] Liao W, Overman MJ, Boutin AT (2019). KRAS-IRF2 Axis Drives Immune Suppression and Immune Therapy Resistance in Colorectal Cancer. Cancer Cell.

[9] Cheng H, Fan K, Luo G (2019). KrasG12D mutation contributes to regulatory T cell conversion through activation of the MEK/ERK pathway in pancreatic cancer. Cancer Lett.

[10] Gruber R, Panayiotou R, Nye E, Spencer-Dene B, Stamp G, Behrens A (2016). YAP1 and TAZ Control Pancreatic Cancer Initiation in Mice by Direct Up-regulation of JAK-STAT3 Signaling. Gastroenterology.

[11] Yamamoto K, Venida A, Yano J (2020). Autophagy promotes immune evasion of pancreatic cancer by degrading MHC-I. Nature.

[12] Blagih J, Zani F, Chakravarty P (2020). Cancer-Specific Loss of p53 Leads to a Modulation of Myeloid and T Cell Responses. Cell Rep.

[13] Duffy MJ, Crown J (2021). Drugging “undruggable” genes for cancer treatment: Are we making progress?. Int J Cancer.

[14] Awad MM, Liu S, Rybkin II (2021). Acquired Resistance to KRASG12C Inhibition in Cancer. N Engl J Med.

[15] O'Sullivan É, Keogh A, Henderson B, Finn SP, Gray SG, Gately K (2023). Treatment Strategies for KRAS-Mutated Non-Small-Cell Lung Cancer. Cancers.

[16] Ramasamy T, Ruttala HB, Munusamy S, Chakraborty N, Kim JO (2022). Nano drug delivery systems for antisense oligonucleotides (ASO) therapeutics. J Controlled Release.

[17] Tran TTT, Phung CD, Yeo BZJ (2024). Customised design of antisense oligonucleotides targeting EGFR driver mutants for personalised treatment of non-small cell lung cancer. eBioMedicine.

[18] Collotta D, Bertocchi I, Chiapello E, Collino M (2023). Antisense oligonucleotides: a novel Frontier in pharmacological strategy. Front Pharmacol.

[19] Wang D, Wang Q, Wang Y (2022). Targeting oncogenic KRAS with molecular brush-conjugated antisense oligonucleotides. Proc Natl Acad Sci.

[20] Ross SJ, Revenko AS, Hanson LL (2017). Targeting KRAS-dependent tumors with AZD4785, a high-affinity therapeutic antisense oligonucleotide inhibitor of KRAS. Sci Transl Med.

[21] Sacco A, Federico C, Todoerti K (2021). Specific targeting of the KRAS mutational landscape in myeloma as a tool to unveil the elicited antitumor activity. Blood.

[22] Rehwinkel J, Gack MU (2020). RIG-I-like receptors: their regulation and roles in RNA sensing. Nat Rev Immunol.

[23] Honda K, Taniguchi T (2006). IRFs: master regulators of signalling by Toll-like receptors and cytosolic pattern-recognition receptors. Nat Rev Immunol.

[24] Besch R, Poeck H, Hohenauer T (2009). Proapoptotic signaling induced by RIG-I and MDA-5 results in type I interferon-independent apoptosis in human melanoma cells. J Clin Invest.

[25] Duewell P, Steger A, Lohr H (2014). RIG-I-like helicases induce immunogenic cell death of pancreatic cancer cells and sensitize tumors toward killing by CD8+ T cells. Cell Death Differ.

[26] Peng B, Nguyen TM, Jayasinghe MK (2022). Robust delivery of RIG-I agonists using extracellular vesicles for anti-cancer immunotherapy. J Extracell Vesicles.

[27] Canon J, Rex K, Saiki AY (2019). The clinical KRAS(G12C) inhibitor AMG 510 drives anti-tumour immunity. Nature.

[28] Usman WM, Pham TC, Kwok YY (2018). Efficient RNA drug delivery using red blood cell extracellular vesicles. Nat Commun.

[29] Jayasinghe MK, Gao C, Yap G (2023). Red Blood Cell-Derived Extracellular Vesicles Display Endogenous Antiviral Effects and Enhance the Efficacy of Antiviral Oligonucleotide Therapy. ACS Nano.

[30] Peng B, Yang Y, Wu Z (2023). Red blood cell extracellular vesicles deliver therapeutic siRNAs to skeletal muscles for treatment of cancer cachexia. Mol Ther.

[31] Petroni G, Buqué A, Coussens LM, Galluzzi L (2022). Targeting oncogene and non-oncogene addiction to inflame the tumour microenvironment. Nat Rev Drug Discov.

[32] Zaanan A, Okamoto K, Kawakami H, Khazaie K, Huang S, Sinicrope FA (2015). The Mutant KRAS Gene Up-regulates BCL-XL Protein via STAT3 to Confer Apoptosis Resistance That Is Reversed by BIM Protein Induction and BCL-XL Antagonism. J Biol Chem.

[33] Munkhbaatar E, Dietzen M, Agrawal D (2020). MCL-1 gains occur with high frequency in lung adenocarcinoma and can be targeted therapeutically. Nat Commun.

[34] DuPage M, Dooley AL, Jacks T (2009). Conditional mouse lung cancer models using adenoviral or lentiviral delivery of Cre recombinase. Nat Protoc.

[35] Schram AM, Gandhi L, Mita MM (2018). A phase Ib dose-escalation and expansion study of the oral MEK inhibitor pimasertib and PI3K/MTOR inhibitor voxtalisib in patients with advanced solid tumours. Br J Cancer.

[36] Adeegbe DO, Liu S, Hattersley MM (2018). BET bromodomain inhibition cooperates with PD-1 blockade to facilitate antitumor response in Kras-mutant non-small cell lung cancer. Cancer Immunol Res.

[37] Yang Z, Liang S-Q, Zhao L (2022). Metabolic synthetic lethality by targeting NOP56 and mTOR in KRAS-mutant lung cancer. J Exp Clin Cancer Res.

[38] Hong DS, Fakih MG, Strickler JH (2020). KRASG12C Inhibition with Sotorasib in Advanced Solid Tumors. N Engl J Med.

[39] Mugarza E, van Maldegem F, Boumelha J (2022). Therapeutic KRASG12C inhibition drives effective interferon-mediated antitumor immunity in immunogenic lung cancers. Sci Adv.

[40] Roberts TC, Langer R, Wood MJA (2020). Advances in oligonucleotide drug delivery. Nat Rev Drug Discov.

[41] Molina-Arcas M, Downward J (2024). Exploiting the therapeutic implications of KRAS inhibition on tumor immunity. Cancer Cell.

[42] Sato Y, Fu Y, Liu H, Lee MY, Shaw MH (2021). Tumor-immune profiling of CT-26 and Colon 26 syngeneic mouse models reveals mechanism of anti-PD-1 response. BMC Cancer.

[43] Luoma AM, Suo S, Wang Y (2022). Tissue-resident memory and circulating T cells are early responders to pre-surgical cancer immunotherapy. Cell.

[44] Bankhead P, Loughrey MB, Fernández JA (2017). QuPath: Open source software for digital pathology image analysis. Sci Rep.

