# Supplementary material for: Combination of KRAS ASO and RIG-I agonist in extracellular vesicles transforms the tumor microenvironment towards effective treatment of KRAS-dependent cancers
Source: Theranostics. 2025 Jun 9;15(14):6818–38. doi: 10.7150/thno.105519 (PMC12203666; doi:10.7150/thno.105519)
Supplement: Supplementary file 1 — Supplementary figures and tables. [file thnov15p6818s1.pdf]

Supporting Information

Combination of KRAS ASO and RIG-I agonist in extracellular vesicles transforms the tumor microenvironment towards effective treatment of KRAS-dependent cancers

Cao Dai Phung[1#], Trinh T.T. Tran[1,13#], Brendon Zhi Jie Yeo[1], Rebecca Carissa Prajogo[1], Evelyn Saudjana[1], Eric Yew Meng Yeo[1],Gao Chang[1], Phuong H.D. Nguyen[1], Migara Kavishka Jayasinghe[1], Xuan T.T. Dang[1], Celest Phang Lixuan[4,5], Trinh Mai Nguyen[4,5], Boya Peng[1], Anh Hong Le[1], Tram T.T Nguyen[1], Gloria Mei En Chan[6], Yuin-Han Loh[7,8,9,11], Boon Cher Goh[1,2], Wai Leong Tam[2,3], Glenn Kunnath Bonney[2], Dahai Luo[4,5], Minh T.N. Le[1,11,12,14,15]*

[1] *Department of Pharmacology and Institute for Digital Medicine, Yong Loo Lin School of Medicine, National University of Singapore*

[2]*Cancer Science Institute of Singapore, National University of Singapore*

[3]*Genome Institute of Singapore, A*STAR*

[4]*Lee Kong Chian School of Medicine, Nanyang Technological University*

[5]*Institute of Structural Biology, Nanyang Technological University*

[6] *Synthetic Biology Translational Research Program, Yong Loo Lin School of Medicine, National University of Singapore*

[7]*Cell Fate Engineering and Therapeutics Laboratory, A*STAR Institute of Molecular and Cell Biology, Singapore, 138673, Singapore*

[8]*Department of Biological Sciences, National University of Singapore, Singapore, 117543, Singapore*

[9]*Integrative Sciences and Engineering Programme (ISEP), NUS Graduate School, National University of Singapore, 21 Lower Kent Ridge Road, Singapore, 119077, Singapore*

[10]*Department of Physiology, Yong Loo Lin School of Medicine, National University of Singapore, Singapore, 117593, Singapore*

[11]*Department of Surgery, Yong Loo Lin School of Medicine, National University of Singapore.*

[12]*Institute of Molecular and Cell Biology, A*STAR, Singapore*

[13] *Vingroup Science and Technology Scholarship Program, Vin University, Hanoi, Vietnam*

[14]*Nanomedicine Translational Research Programme, Yong Loo Lin School of Medicine, National University of Singapore*

[15]*Cancer Translational Research Programme, Yong Loo Lin School of Medicine, National University of Singapore*

C.D. Phung and T.T.T. Tran contributed equally to this work

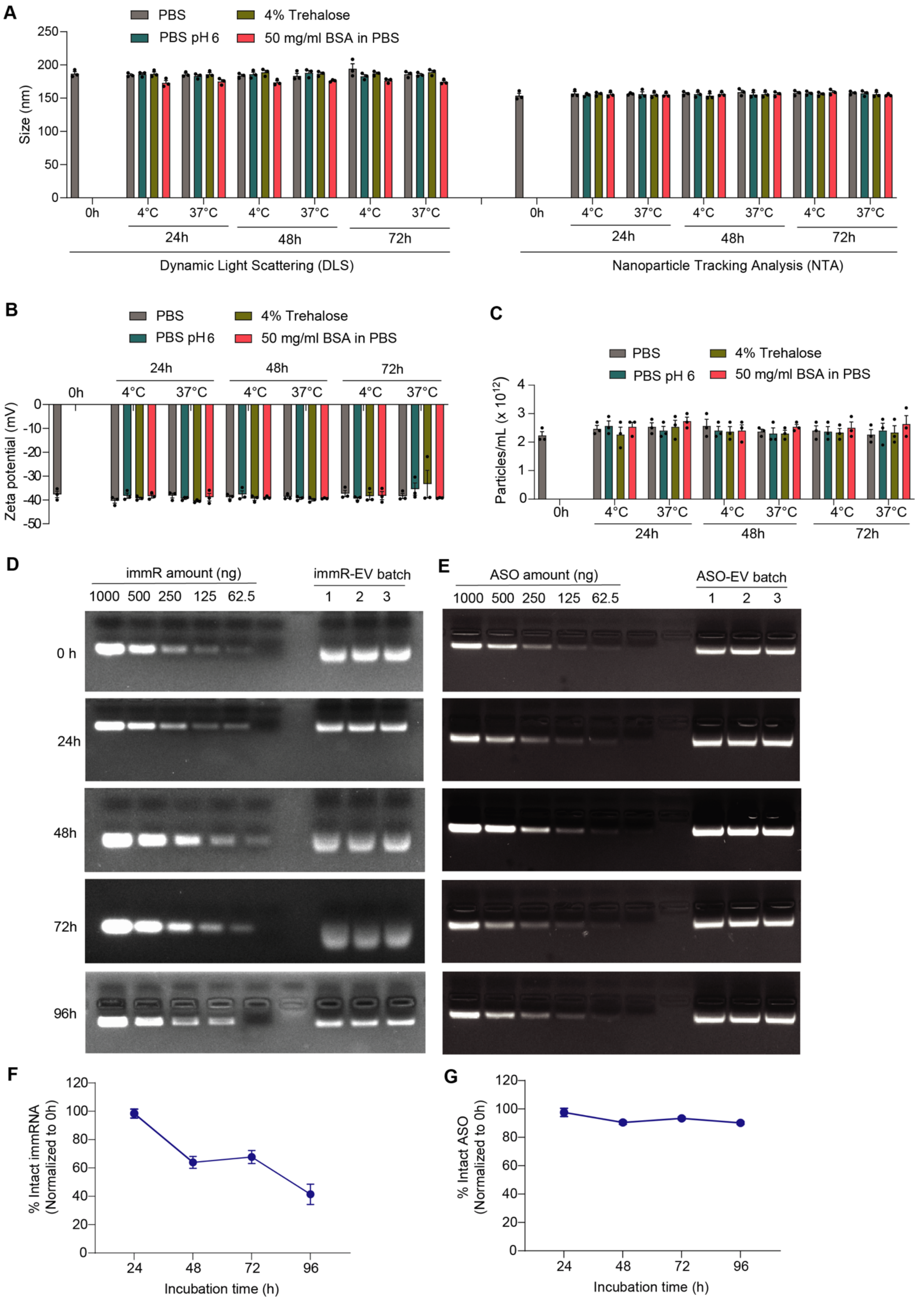
A
PBS
4% Trehalose
PBS pH 6
50 mg/ml BSA in PBS
Size (nm)
0h
4°C
37°C
24h
48h
72h
Dynamic Light Scattering (DLS)
Nanoparticle Tracking Analysis (NTA)
B
Zeta potential (mV)
C
Particles/mL (x 10^12)
D
immR amount (ng)
1000 500 250 125 62.5
immR-EV batch
1 2 3
0 h
24h
48h
72h
96h
E
ASO amount (ng)
ASO-EV batch
F
% Intact immRNA (Normalized to 0h)
Incubation time (h)
G
% Intact ASO (Normalized to 0h)

Figure S1. **Stability of RBCEVs**. (**A**) Particle size, (**B**) zeta potential, and (**C**) concentration of RBCEVs measured by dynamic light scattering (DLS) or nanoparticle tracking analysis (NTA) method after incubating in different buffers, temperatures, or incubation time. (**D**-**E**) Agarose gel electrophoresis analysis of (**D**) immRNA and (**E**) ASO stability in RBCEVs after storing in -80 ^{o}C at different time points. (**F**-**G**) Quantification of intact (**F**) immRNA and (**G**) ASO percentages in RBCEVs (normalized to 0h) at storing conditions as described in (**D**-**E**). The graphs present mean ± SEM.

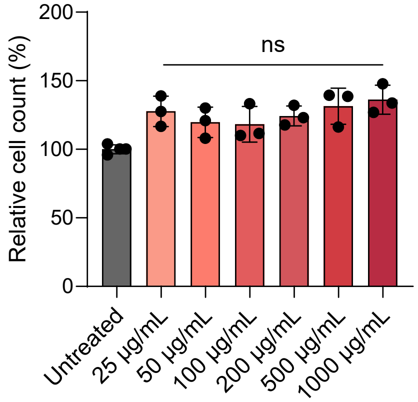

Figure S2. Relative cell counts of A427 cells two days after treatment with different doses of RBCEVs determined by the CCK8 assay (n = 3 biological repeats). The graphs present mean ± SEM. Ns – not significant, determined using One-Way ANOVA test.

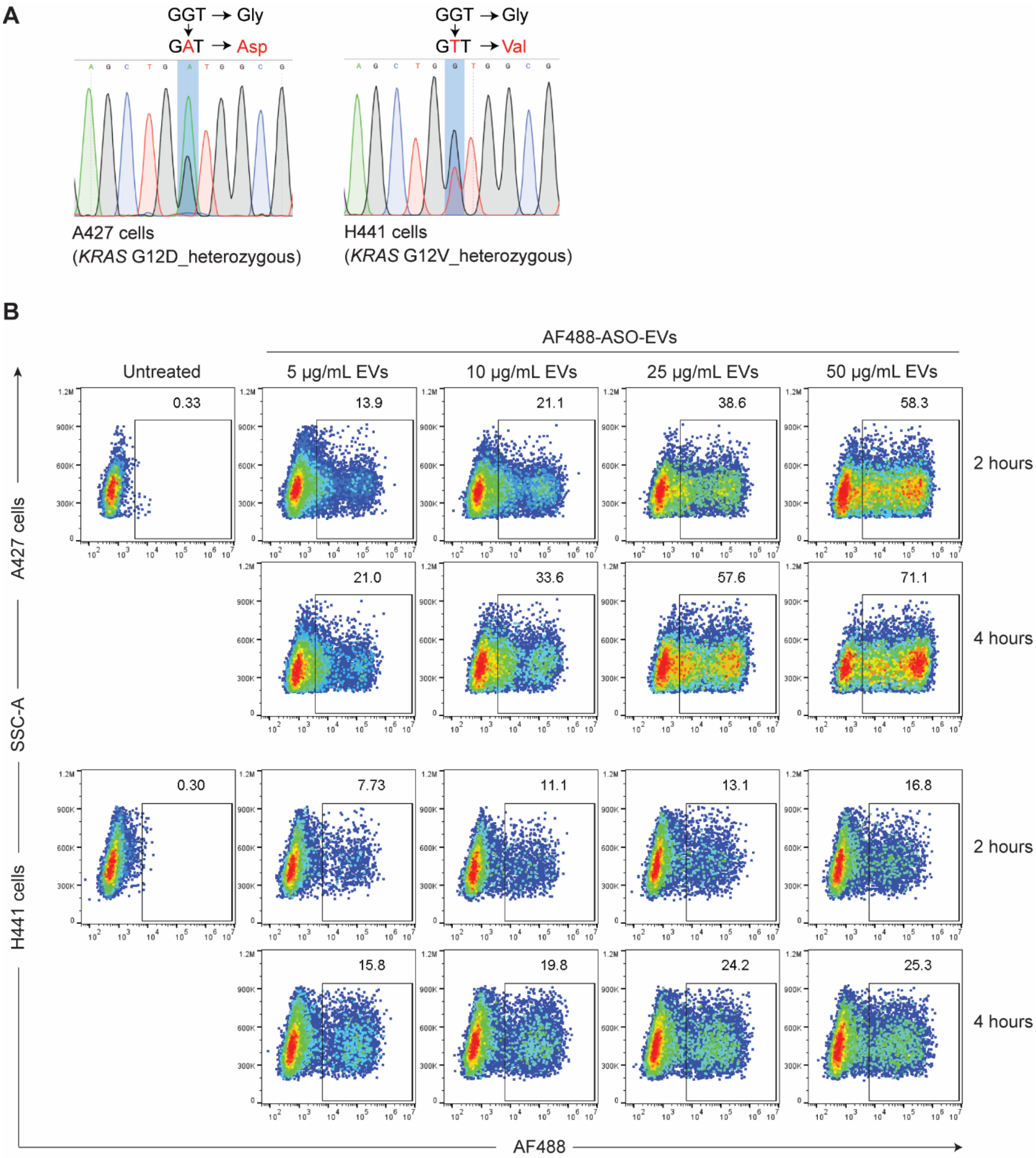

Figure S3. **Uptake of RBCEVs by *KRAS*-mutated cancer cells *in vitro.*** (**A**) Sanger sequencing for identifying *KRAS* mutation status in A427 and H441 cancer cells. (**B**) Flow cytometric analysis of AF488-ASO-loaded RBCEVs taken up by A427 and H441 cells after 2 h of incubation at different concentrations.

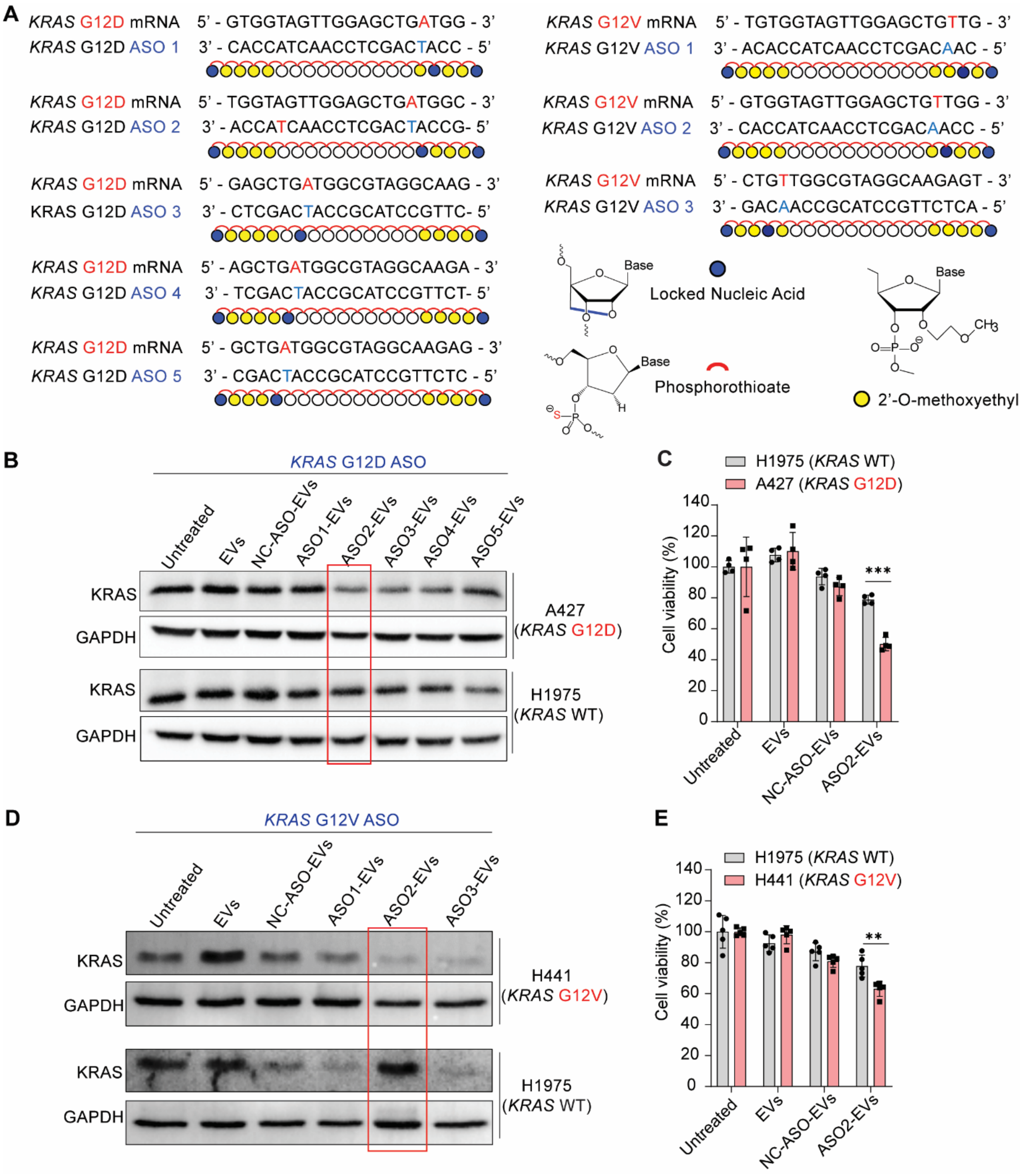

Figure S4. **Antisense oligonucleotides (ASOs) are designed to specifically inhibit *KRAS* mutants**. (**A**) Sequences and chemical modifications of anti-*KRAS* G12D and anti-*KRAS* G12V ASOs. (**B**) Knockdown of KRAS in A427 cells bearing *KRAS* G12D mutation and H1975 cells with wild-type *KRAS* two days following treatment with *KRAS* G12D ASO-loaded RBCEVs determined by Western blot analysis. (**C**) Viability of A427 and H1975 cells two days following treatment with RBCEVs loaded with *KRAS* G12D ASO2 or NC ASO determined by CCK-8 assay (n = 5). (**D**) Knockdown of KRAS in H441 cells bearing *KRAS* G12V mutation and H1975 cells with wild-type *KRAS* two days following treatment with *KRAS* G12V ASO-loaded RBCEVs determined by Western blot analysis. (**E**) Viability of H441 and H1975 cells two days following treatment with RBCEVs loaded with *KRAS* G12V ASO2 or NC ASO determined by CCK-8 assay (n = 5).

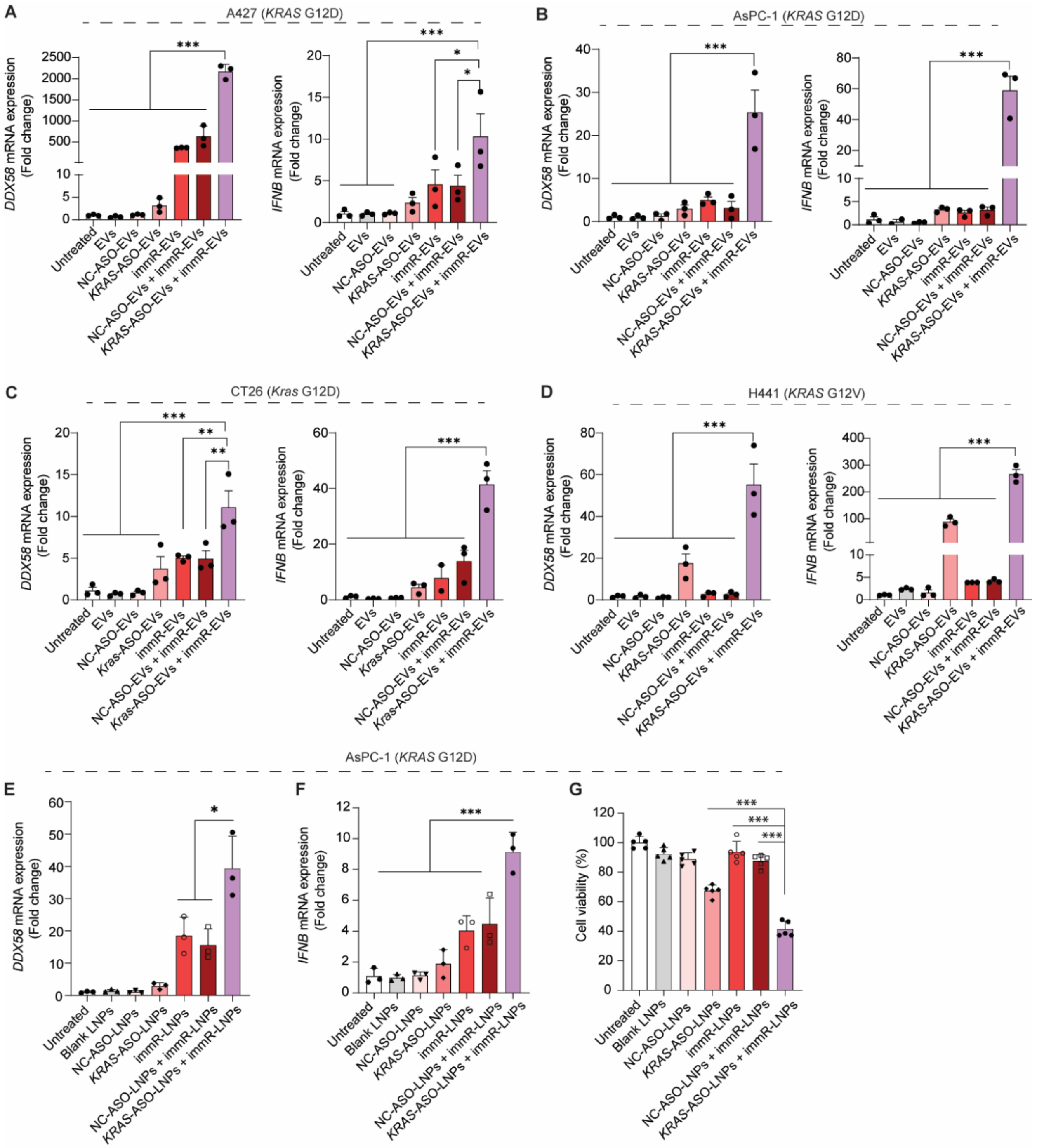

Figure S5. **Combination of ASOs targeting *KRAS* mutants with immunomodulatory RNA synergistically activates the pro-inflammatory cytokine signaling in cancer cells.** qPCR analysis of *DDX58* and *IFNB* expression in (**A**) A427 cells (*KRAS* G12D), (**B**) H441 cells (*KRAS* G12V), (**C**) CT26 cells (*Kras* G12D), and (**D**) H441 cells (*KRAS* G12V) cells after treatments with different RBCEV formulations for 48 h. (**E-F**) qPCR analysis of expression of (**E**) *DDX58* and (**F**) *IFNB* in AsPC-1 cells after treatments with different lipid nanoparticle (LNP) formulations for 48 h. (**J**) Cell viability of AsPC-1 cells after treatments with different LNP formulations for 48 h determined by CCK-8 assay.

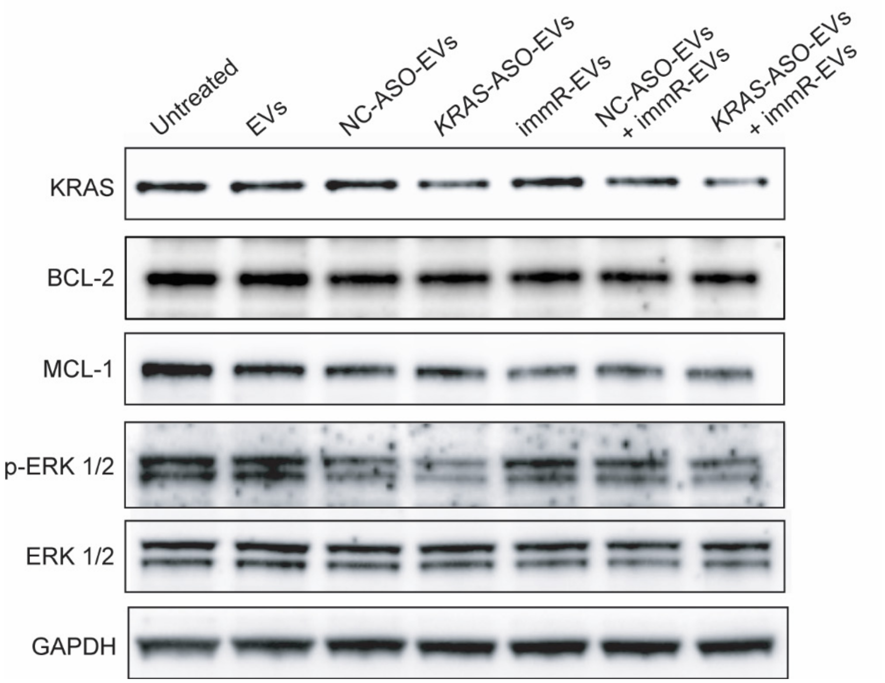

Figure S6. Western blot analysis of KRAS, BCL-2, MCL-1, p-ERK 1/2, and ERK 1/2 protein expression in A427 cells after the treatment with different RBCEV formulations for 48 h.

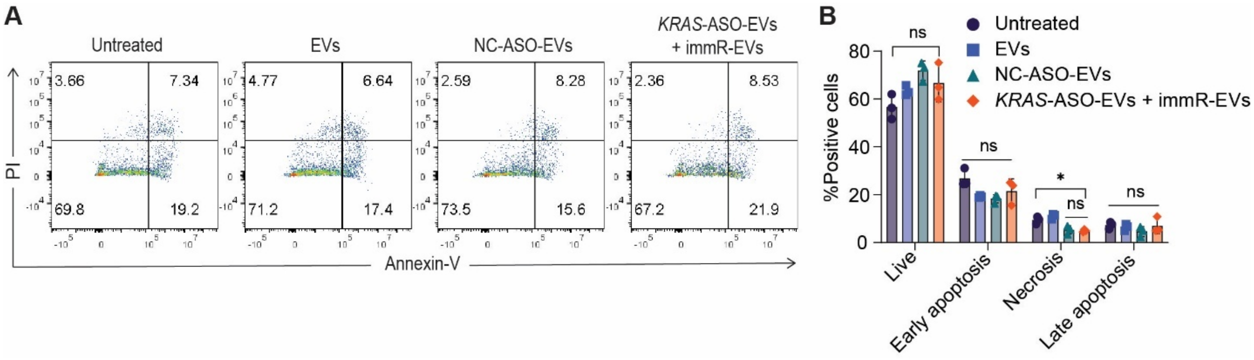

Figure S7. (**A**) Flow cytometric analysis of Annexin V/PI staining in human bronchial epithelial cells (HBECs) cells 24 h post-treatment with 50 µg/mL unloaded RBCEVs (EVs) or 50 µg/mL RBCEVs loaded with either NC ASO (NC-ASO-EVs), or combined *KRAS* G12D ASO and immRNA (*KRAS*-ASO-EVs + immR-EVs). (B) Proportion of live (Annexin-, PI-) early apoptotic (Annexin+, PI-), necrotic (Annexin-, PI+), and late apoptotic (Annexin+, PI+) HBECs treated as described in (A) (n = 3). The graphs present mean ± SEM. Ns – not significant, determined using One-Way ANOVA test.

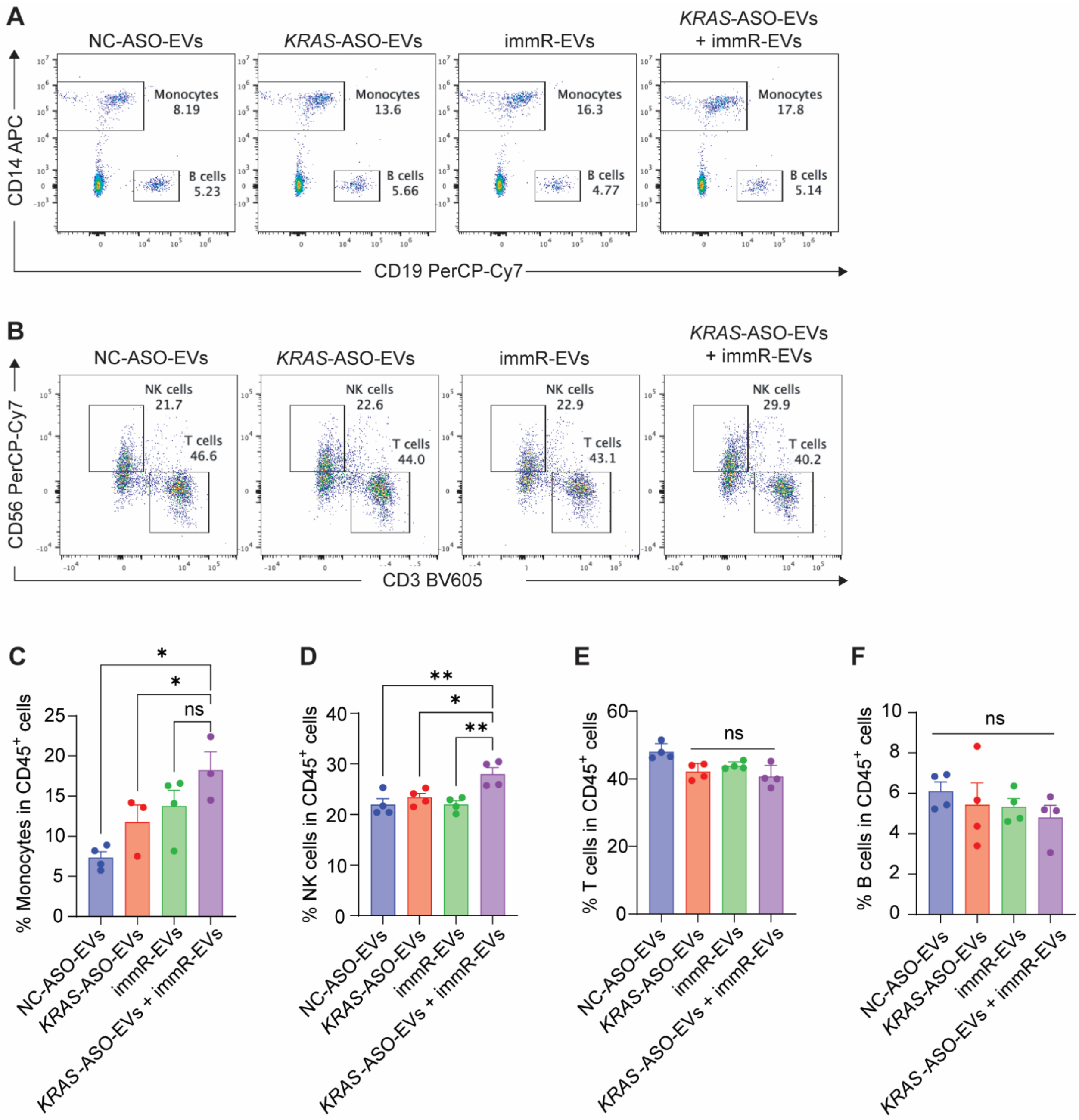

Figure S8. (**A-B**) Flow cytometric analysis of monocytes, B cells, NK cells, and T cells infiltrated into spheroids formed by A427 cells 48 h after the co-culture of treated spheroids and peripheral blood mononuclear cells (PBMCs). (**C-F**) Quantification of percentages of (**C**) monocytes, (**D**) NK cells, (**E**) T cells, and (**F**) B cells in total $CD45^+$ cells infiltrated into treated spheroids as shown in (A-B). The graphs present mean ± SEM. **P < 0.01, and ***P < 0.001, determined using One-Way ANOVA test.

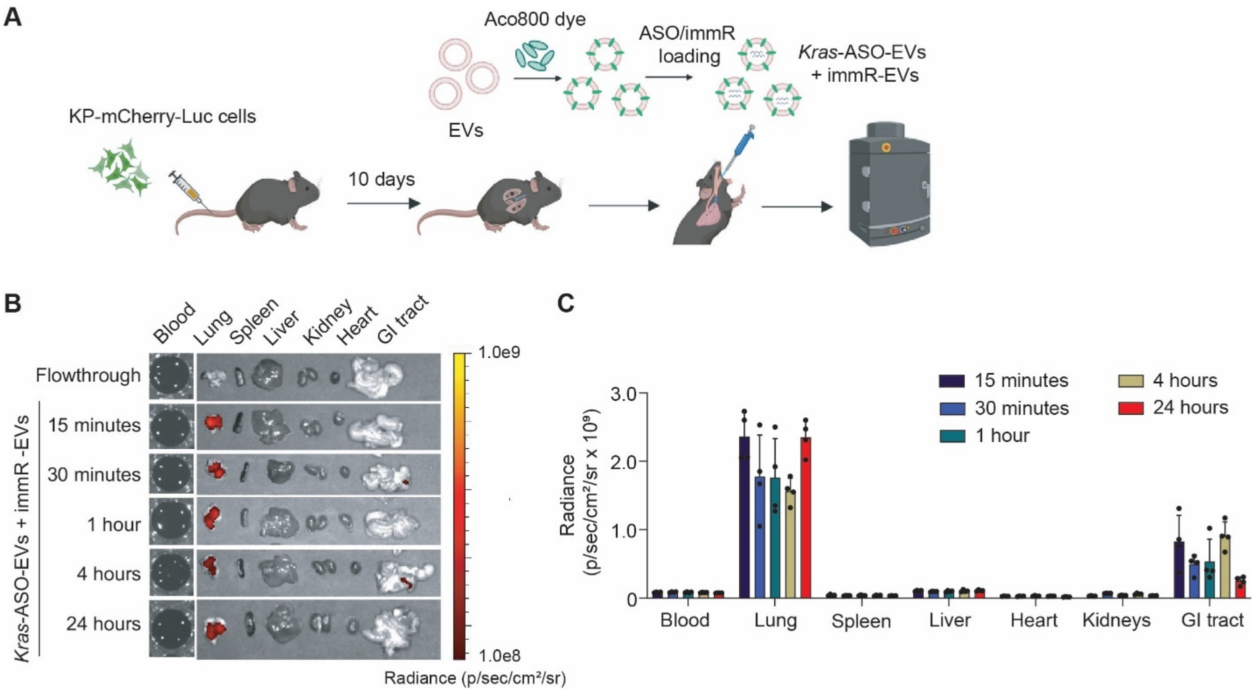

Figure S9. **Biodistribution of RBCEVs loaded with ASO and immRNA into orthotopic lung cancer model after intratracheal administration**. (**A**) Schematic intratracheal delivery of Aco800 dye-labeled RBCEVs loaded with *Kras* ASO and immRNA in C57BL/6 mice bearing lung tumors generated by injection of KP-mCherry-Luc cells in the tail vein. (**B**) Fluorescent images of organs from C57BL/6 mice administered with Aco800-labeled RBCEVs loaded with *Kras* ASO and immRNA by intratracheal administration. Images were captured at different time points after the administration using an IVIS instrument. Color scale represents the Aco800 dye signals. (**C**) Total radiant efficiency of Aco800 fluorescence in various organs of C57BL/6 mice at different time points post-administration (n = 3).

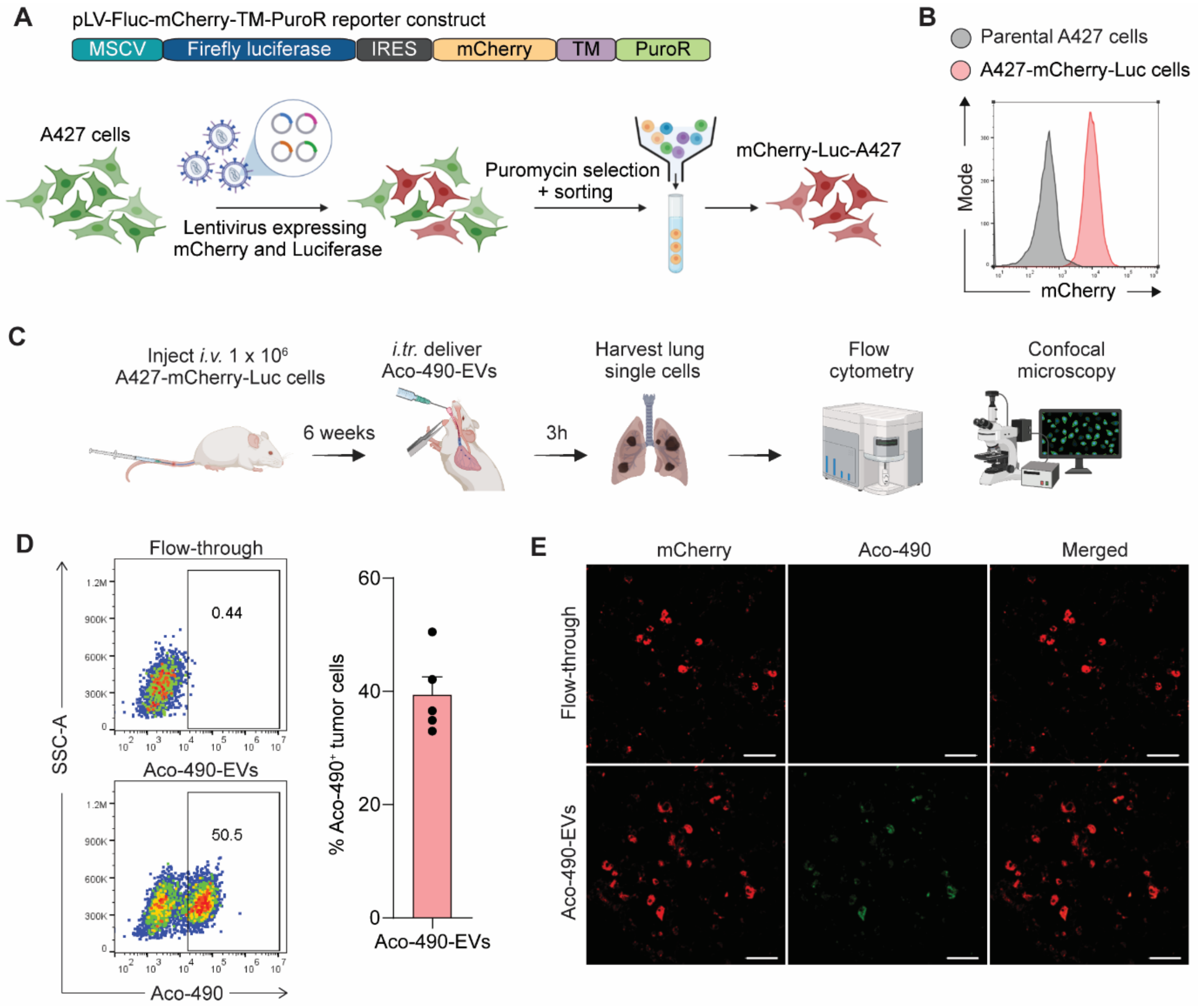

Figure S10. **RBCEVs are effectively taken up by KRAS-mutated tumor cells *in vivo***. (**A**) Construct of pLV-Fluc-mCherry-TM-PuroR plasmid and schematic for generating stable mCherry and Luciferase-expressing A427 lung cancer cells (A427-mCherry-Luc). (**B**) Flow cytometric analysis of mCherry protein expression in A427-mCherry-Luc tumor cells. (**C**) Schematic intratracheal delivery of RBCEVs in NSGS mice bearing lung tumors which were generated by injection of A427-mCherry-Luc cells in the tail vein. (**D**) Flow cytometric and analysis of Aco-490-labeled EV signal in tumor cells in the lungs of A427 tumor-bearing mice treated with Aco-490-labeled RBCEVs (n = 5). (**E**) Confocal microscopy analysis of Aco-490-labeled RBCEVs (green) taken up by A427-mCherry-Luc cells (red) in the lung after intratracheal administration. Scale bar: 50 μm.

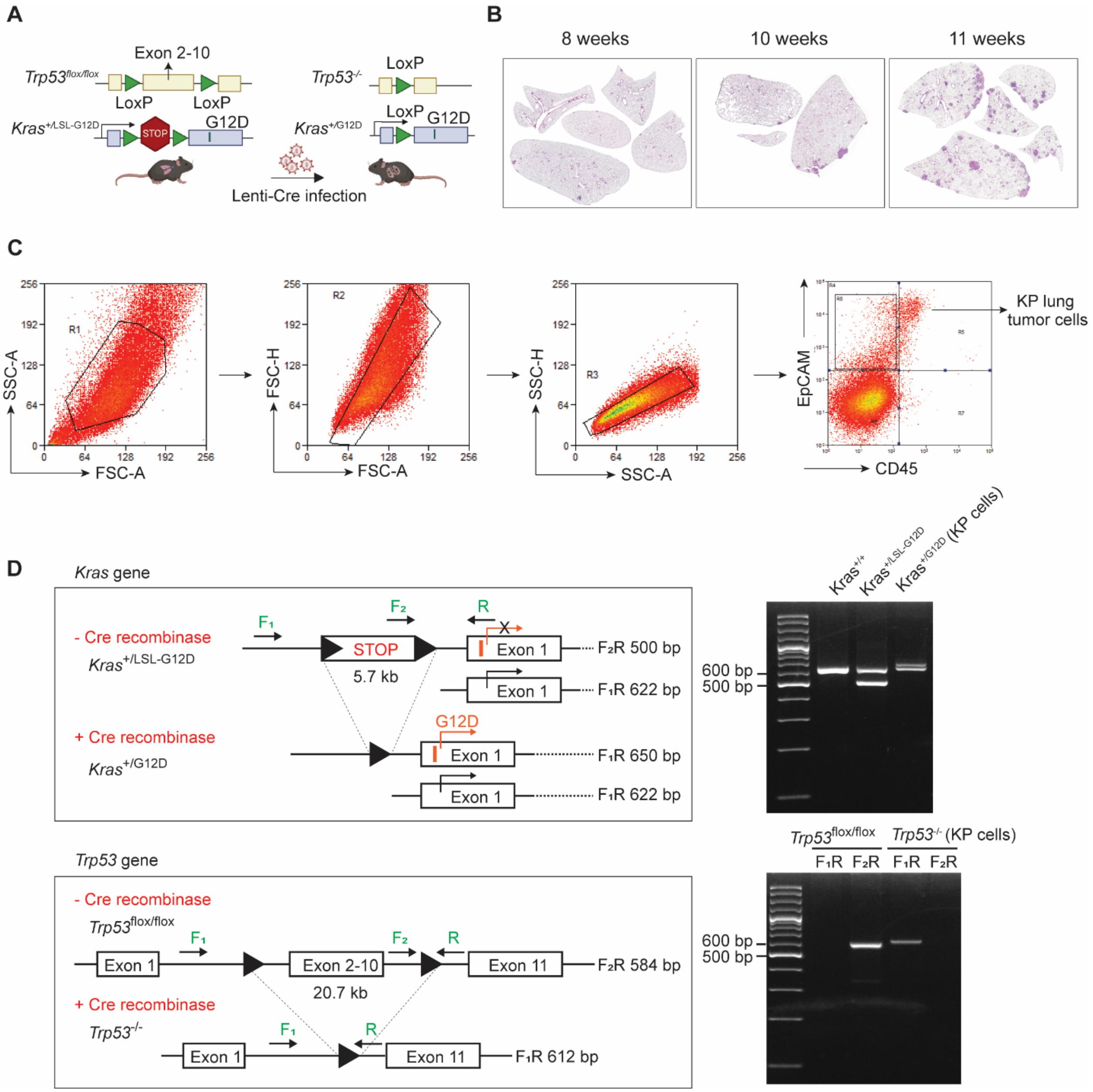

Figure S11. **Induction of non-small cell lung cancer in a genetically engineered mouse model with *Kras* G12D mutation**. (**A**) Schematic of the tumor induction in Kras$^{LSL-G12D}$/+; p53$^{fl/fl}$ (KP) mouse model. Cre recombinase-expressing lentivirus (Lenti-Cre) was delivered intratracheally to KP mice. (**B**) Haematoxylin and eosin (H&E) stained lungs from KP mice 8, 10, and 11 weeks after infection with Lenti-Cre. Scale bar: 200 µm. (**C**) FACS gating strategy for sorting EpCAM^{+}CD45^{-} tumor cells (KP tumor cells) from the lung of KP mice. (**D**) Schematic of primers designed for the detection of wild-type (*Kras*$^{+/+}$), heterozygous (*Kras*$^{+/LSL-G12D}$) and recombined (*Kras*$^{+/G12D}$) *Kras* genotypes, and exon 2-10 deletion in the *Trp53* gene (left panels). (Top right panel) Agarose gel electrophoresis of *Kras* PCR products from wild-type *Kras* control cells (lane 1), heterozygous control cells (lane 2) and KP cells (lane 3) with all 3 primers being added into each PCR reaction tube. (Bottom right panel) Agarose gel electroporation of *Trp53* PCR products when employing different primer pairs in the *Trp53*$^{f/f}$ control cells (lane 1 and 2) and KP cells (lane 3 and 4). Either F1 and R primer pair (lane 1 and 3) or F2 and R primer pair (lane 2 and 4) were added to the PCR reactions of each cell type.

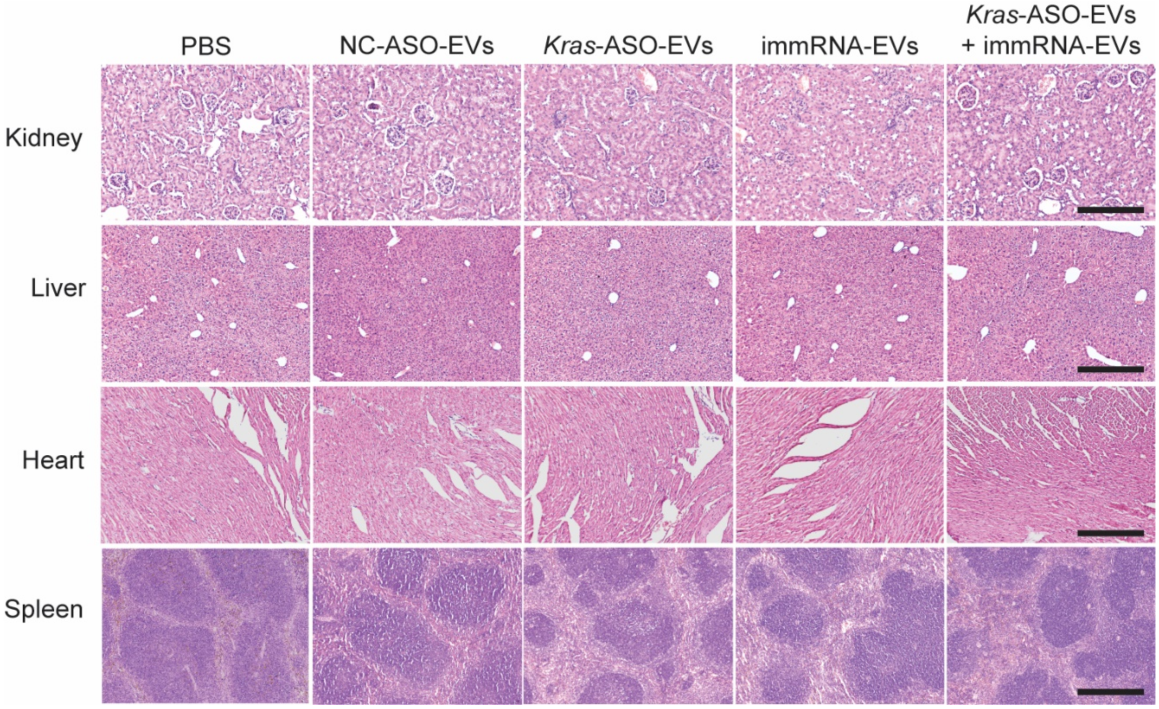

Figure S12. Histological analysis of major organs collected from Kras$^{LSL\text{-}G12D}$/+; p53$^{fl/fl}$ (KP) mice after intratracheal administration of PBS or 10 mg/kg RBCEVs loaded with either NC ASO (NC-ASO-EVs), *Kras* G12D ASO (*Kras*-ASO-EVs), immRNA (immR-EVs), or combined *Kras* G12D ASO and immRNA (*Kras*-ASO-EVs + immR-EVs) every three days for three weeks. Scale bar: 200 μm.

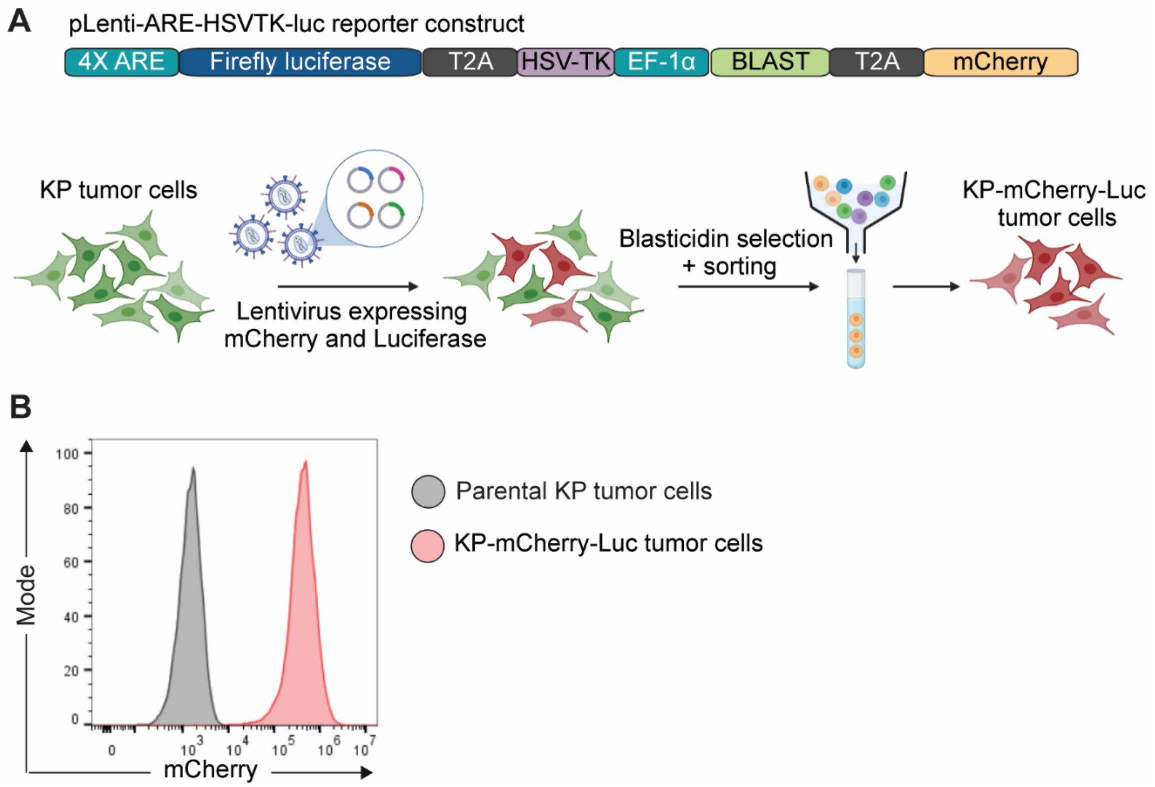

Figure S13. **Generation of mCherry and luciferase-expressing KP tumor cells.** (**A**) Construct of pLenti-ARE-HSVTK-luc plasmid and schematic for generating stable luciferase and mCherry-expressing KP tumor cells (mCherry-Luc-KP tumor cells). (**B**) Flow cytometric analysis of mCherry protein expression in mCherry-Luc-KP tumor cells.

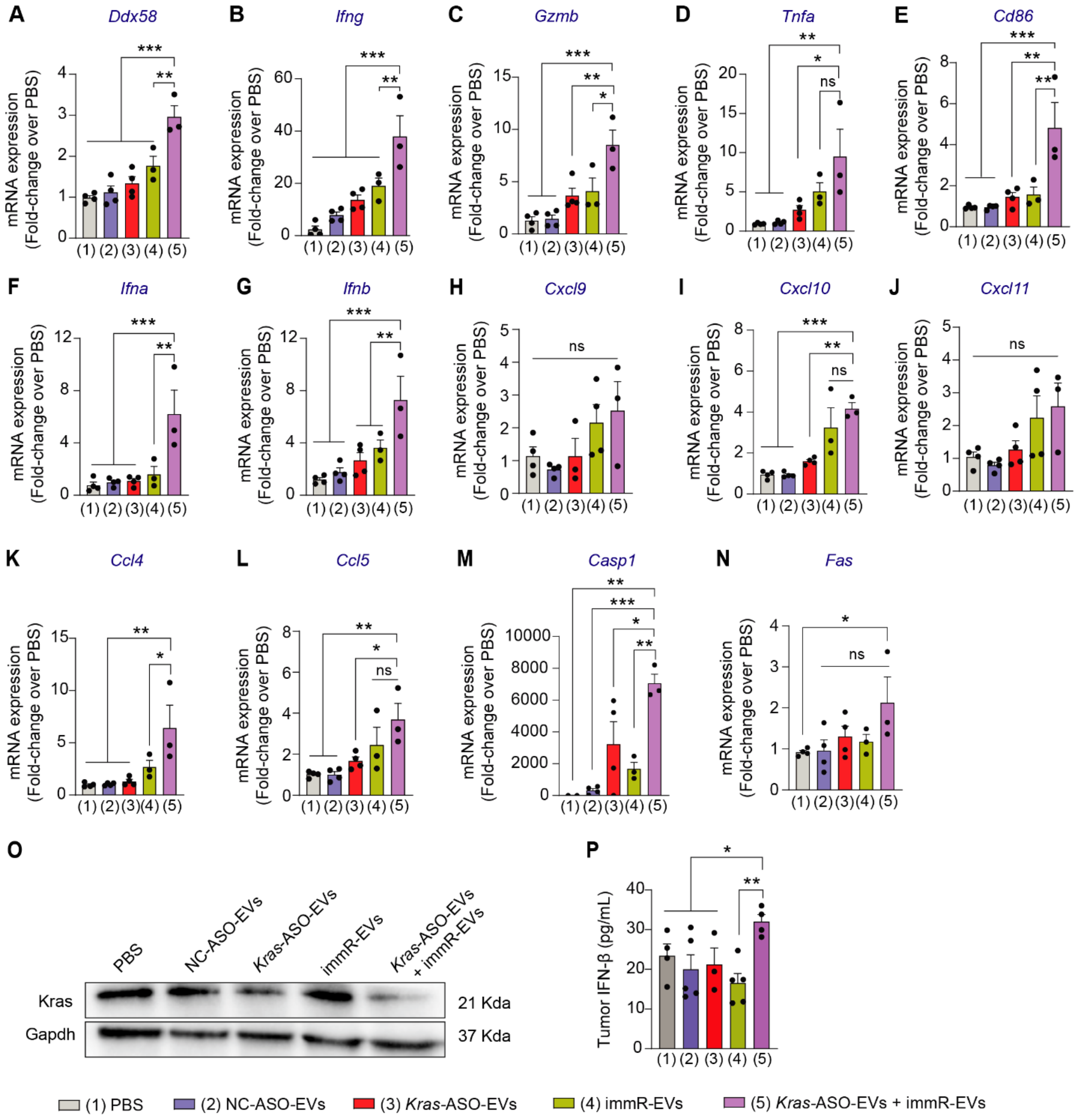

Figure S14. **Analysis of gene and protein expression in CT26 tumors.** (**A-N**) Statistical comparisons of gene expression in the CT26 tumors intratumorally administered with PBS or 5 mg/kg of NC-ASO-EVs, *Kras* G12D ASO (*Kras*-ASO-EVs), immRNA (immR-EVs), or combined *Kras* G12D ASO and immRNA (*Kras*-ASO-EVs + immR-EVs) every three days. (**O**) Western blot analysis of Kras protein expression in the CT26 tumors two days after intratumorally injecting with one dose of PBS or 5 mg/kg of NC-ASO-EVs, *Kras* G12D ASO (*Kras*-ASO-EVs), immRNA (immR-EVs), or combined *Kras* G12D ASO and immRNA (*Kras*-ASO-EVs + immR-EVs). (**P**) IFN-β concentration in treated CT26 tumor lysates measured by ELISA. Data represented as mean ± SEM. The graphs present the mean ± SEM. *P <0.05, **P < 0.01, ***P < 0.001 determined by One-Way ANOVA test.

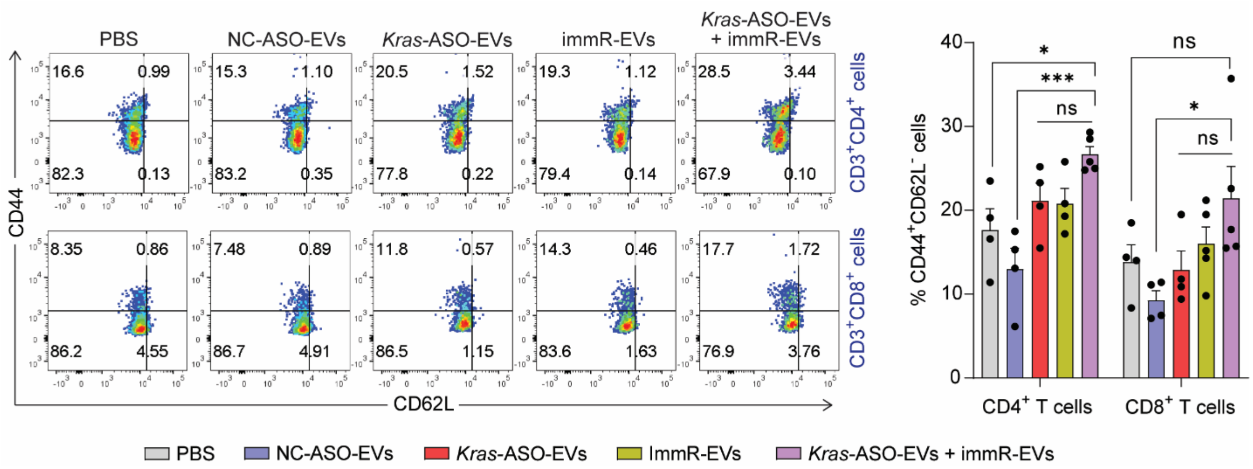

Figure S15. **Analysis of effector memory T cells in the orthotopic lung tumor with mCherry-Luc-KP tumor cells in C57BL/6 mice**. Representative flow cytometry plot (left) and quantification (right) of effector memory T cells ($CD44^{+}CD62L^{-}$) in the spleen of treated mice at the end of the study. The graphs present the mean ± SEM. Ns – not significant, *P <0.05, **P < 0.01, ***P < 0.001 determined by One-Way ANOVA test.

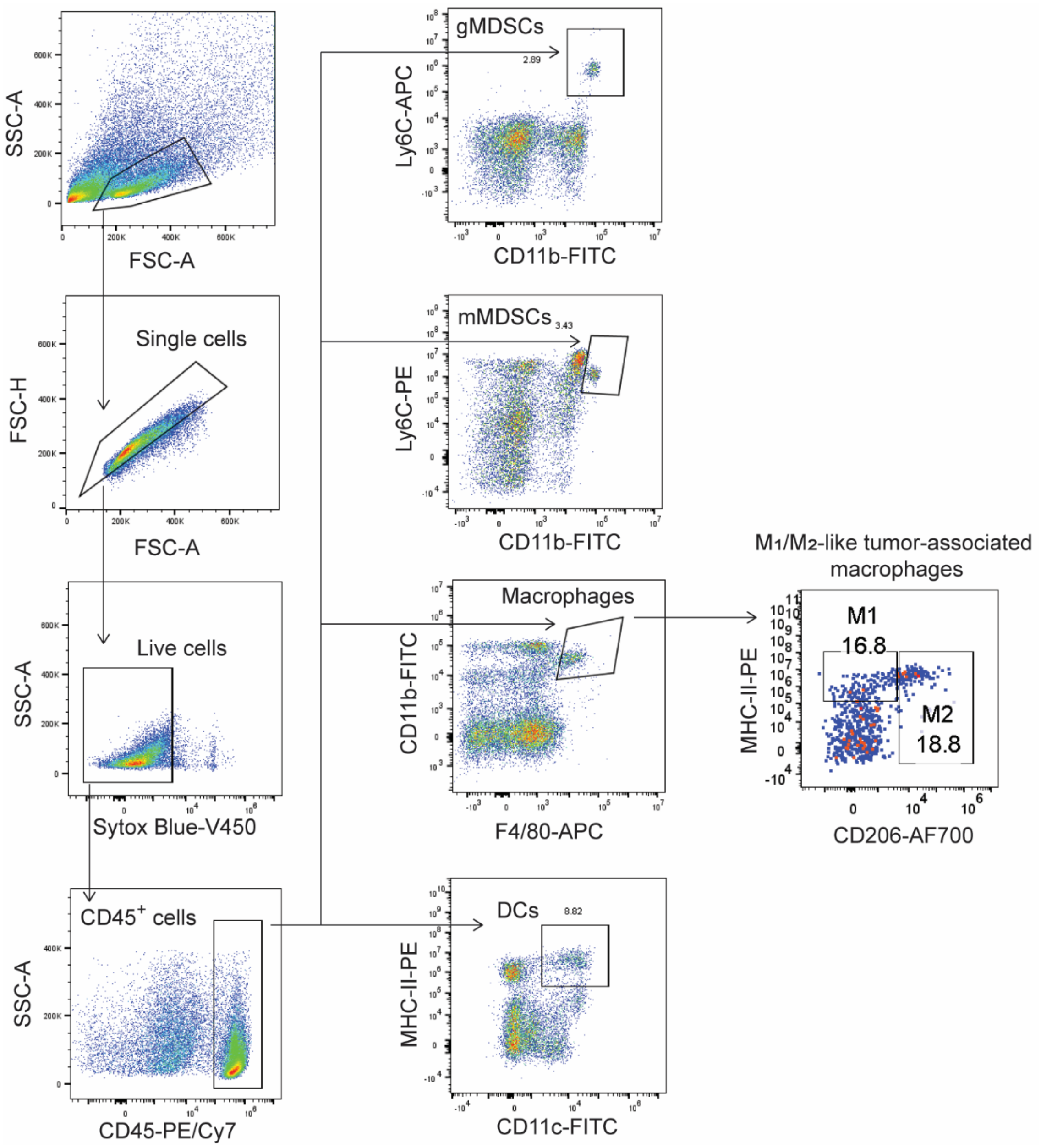

Figure S16. Gating scheme for flow cytometric analysis of immune cell populations in the lung of the orthotopic KP tumor lung cancer model. Cells were stained separately with distinct panels of antibody-conjugated fluorophores for analysis.

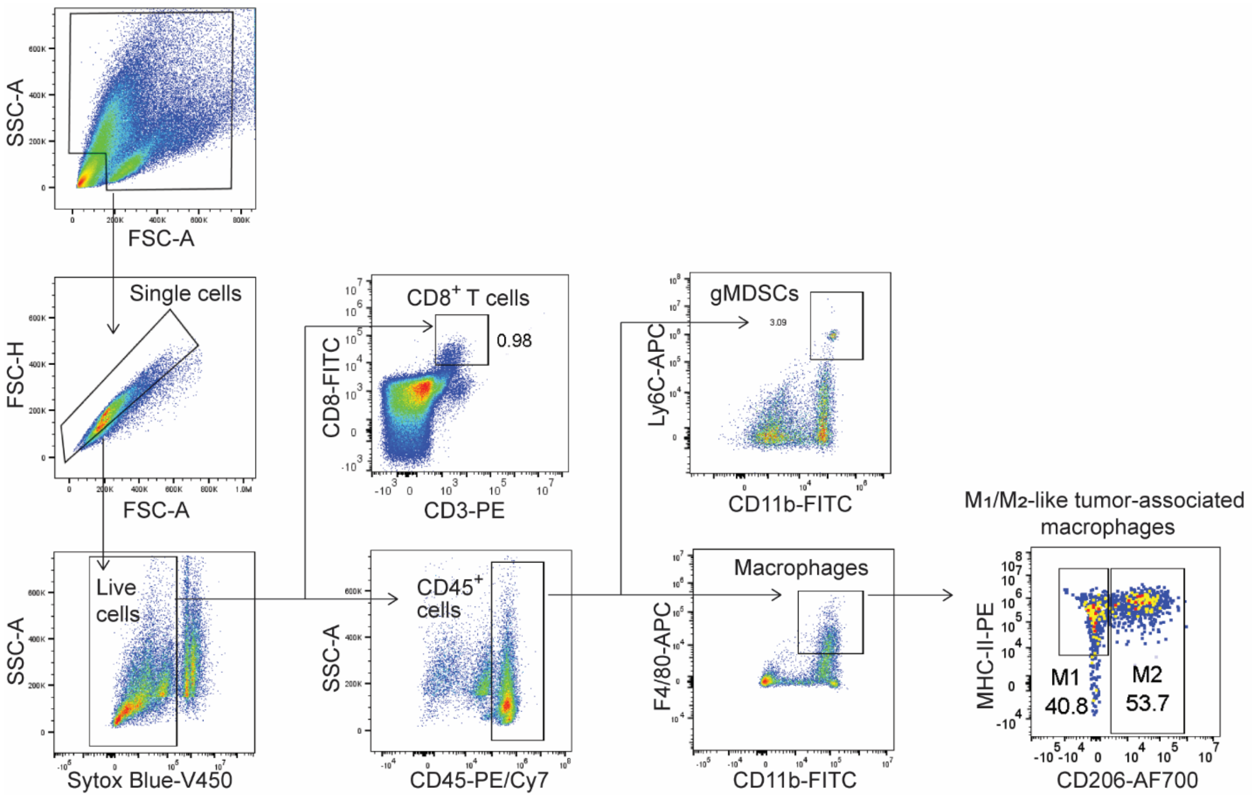

Figure S17. Gating scheme for flow cytometric analysis of immune cell populations in the CT26 tumor. Cells were stained separately with distinct panels of antibody-conjugated fluorophores for analysis.

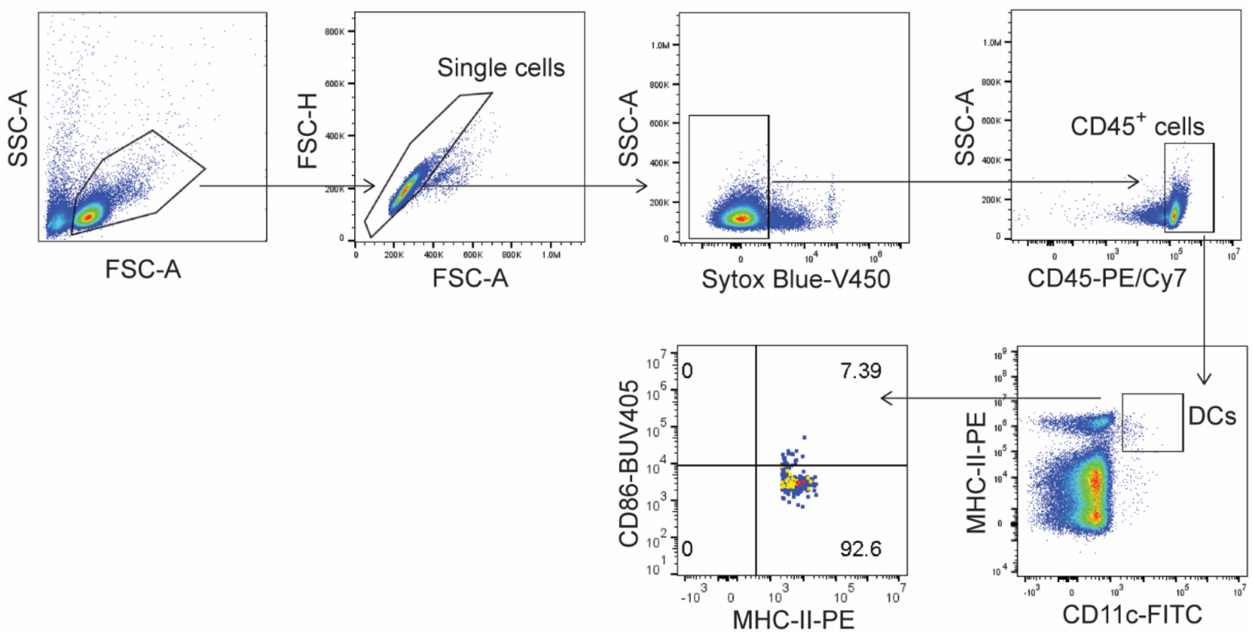

Figure S18. Gating scheme for flow cytometric analysis of dendritic cell activation in the tumor-draining lymph node of treated CT26 tumor-bearing mice at the end of the study.

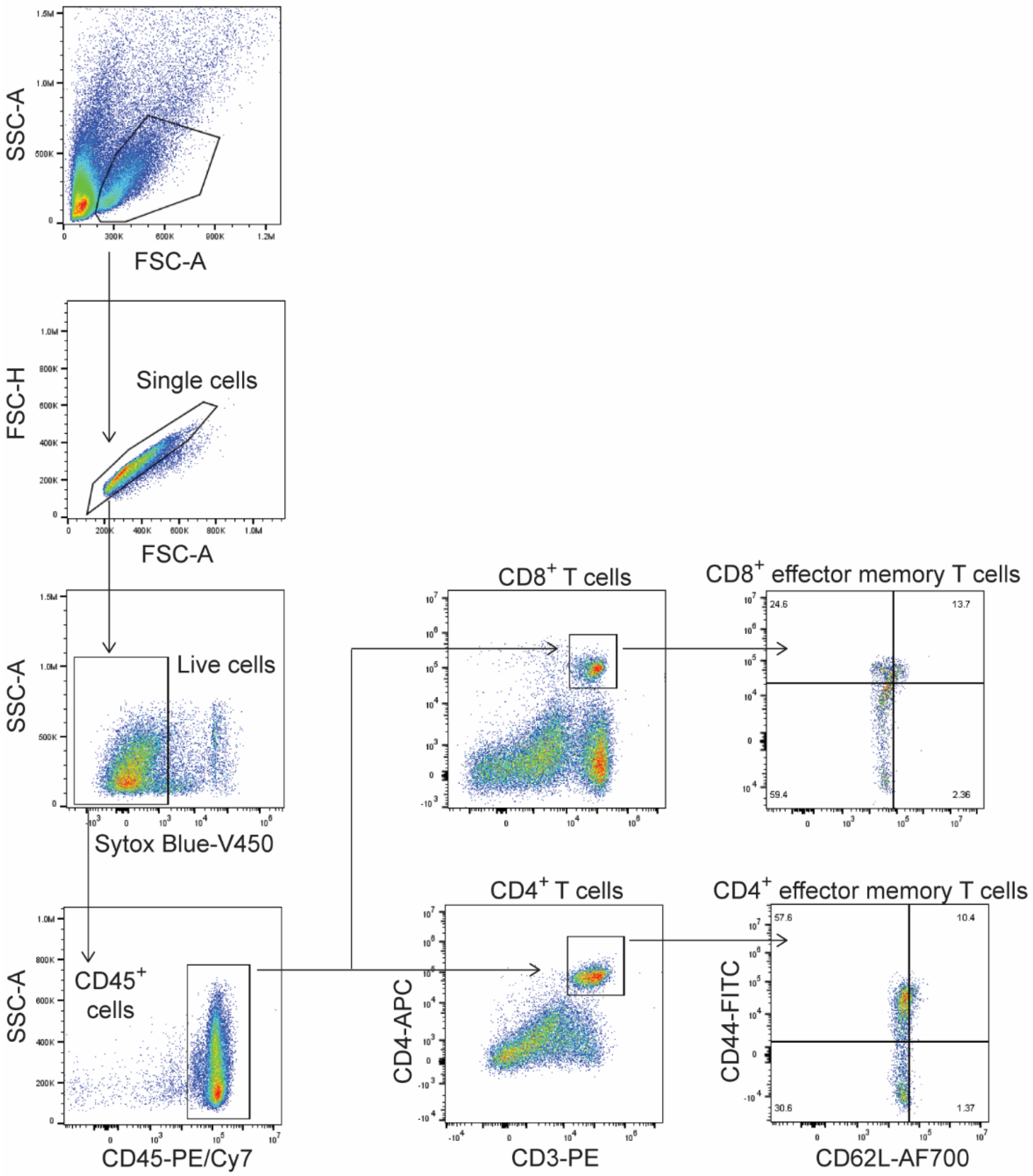

Figure S19. Gating scheme for flow cytometric analysis of effector memory T cells in the spleen of treated CT26 tumor-bearing mice and orthotopic KP lung tumor-bearing mice at the end of the study. Cells were stained separately with distinct panels of antibody-conjugated fluorophores for analysis.

Table S1. Primer list for KP mouse genotyping

Target genes	Direction	Primer sequence
Kras	*Primer1-F*	GTCTTTCCCCAGCACAGTGC
	Primer2-R	CTCTTGCCTACGCCACCAGCTC
	Primer3-F	AGCTAGCCACCATGGCTTGAGTAAGTCTGCA
Trp53	*Primer1-F*	CACAAAAACAGGTTAAACCCA
	Primer2-F	AAGGGGTATGAGGGACAAGG
	Primer3-R	GAAGACAGAAAAGGGGAGGG

Table S2. Primer list for Sanger sequencing

Target genes	Direction	Primer sequence
KRAS	*Exon2-F*	GGTGGAGTATTTGATAGTGTATTAACC
	Exon2-R	AGAATGGTCCTGCACCAGTAA

Table S3. Primer list for RT-qPCR analysis

Genes	Forward sequence	Reverse sequence
**Human**		
GAPDH	GGAGCGAGATCCCTCCAAAAT	GGCTGTTGTCATACTTCTCATGG
KRAS	CAGTAGACACAAAACAGGCTCAG	TGTCGGATCTCCCTCACCAATG
DDX58	GCCATTACACTGTGCTTGGAGA	CCAGTTGCAATATCCTCCACCA
IFNB	CTCTCCTGTTGTGCTTCTCC	GTCAAAGTTCATCCTGTCCTTG
**Mouse**		
Gapdh	AGGTCGGTGTGAACGGATTTG	TGTAGACCATGTAGTTGAGGTCA
Kras	GGAGTACAGTGCAATGAGGGAC	CCAGGACCATAGGCACATCTTC
Ddx58	GAG AGT CAC GGG ACC CAC T	CGG TCT TAG CAT CTC CAA CG
Ido-1	ACACGAGGCTGGCAAAGAA	TGACAAACTCACGGACTGGG
Ifng	CGGCACAGTCATTGAAAGCCTA	GTTGCTGATGGCCTGATTGTC
Gzmb	TGTTTTCTCTGCCATCTGCTCTC	GCTTTGTAAAAGTCTCCAGCCTGTG
Tnf-α	CCCTCACACTCAGATCATCTTCT	GCTACGACGTGGGCTACAG
Il-12	GATGACATGGTGAAGACGGC	AGGCACAGGGTCATCATCAA
Il-6	CCTCTGGTCTTCTGGAGTACC	ACTCCTTCTGTGACTCCAGC
Il-15	GTGACTTTCATCCCAGTTGC	TTCCTTGCAGCCAGATTCTG
Cd80	CCTCAAGTTTCCATGTCCAAGGC	GAGGAGAGTTGTAACGGCAAGG
Cd86	ACGTATTGGAAGGAGATTACAGCT	TCTGTCAGCGTTACTATCCCGC
Ifna	GAGAAGAAACACAGCCCCTG	TCAGTCTTCCCAGCACATTG
Ifnb	AACCTCACCTACAGGGCGGACTTCA	TCCCACGTCAATCTTTCCTCTTGCTTT
Cxcl10	GACGGTCCGCTGCAACTG	CTTCCCTATGGCCCTCATTC T
Cxcl11	AACAGGAAGGTCACAGCCATAGC	TTTGTCGCAGCCGTTACTCG
Cxcl9	CCTAGTGATAAGGAATGCACGATG	CTAGGCAGGTTTGATCTCCGTTC
Ccl4	ACCCTCCCACTTCCTGCTGTTT	CTGTCTGCCTCTTTTGGTCAGG

Ccl5	CCTGCTGCTTTGCCTACCTCTC	ACACACTTGGCGGTTCCTTCGA
Ccl9	TCCAGAGCAGTCTGAAGGCACA	CCGTGAGTTATAGGACAGGCAG
Cd69	GGGCTGTGTTAATAGTGGTCCTC	CTTGCAGGTAGCAACATGGTGG
Cd96	CATGACAGCTTGGTGTATGGCTC	CAGTGGGTAGATGTTTCGTTGGG
IL-1b	TGGACCTTCCAGGATGAGGACA	GTTCATCTCGGAGCCTGTAGTG
Casp1	GGCACATTTCCAGGACTGACTG	GCAAGACGTGTACGAGTGGTTG
Fas	CTGCGATTCTCCTGGCTGTGAA	CAACAACCATAGGCGATTTCTGG
Pdl1	TGCGGACTACAAGCGAATCACG	CTCAGCTTCTGGATAACCCTCG
Ifna	AAGACTGAGTGAGAAGGAGTGAG	GAGATGCCAGAATTTGAGCAGTG

Table S4. Antibody list for immunoassays

Antibody	Company	Catalog No.	Dilution
BAND 3	Santa Cruz	sc-133190	1:1000
STOMATIN	Santa Cruz	sc-376869	1:1000
ALIX	Santa Cruz	sc-53538	1:500
TSG101	Santa Cruz	sc-7964	1:500
HBA	Santa Cruz	sc-21005	1:1000
GPA	Biolegend	306602	1:500
CALNEXIN	Santa Cruz	sc-23954	1:500
β-ACTIN	Proteintech	HRP-60008	1:5000
GAPDH	Proteintech	HRP-60004	1:5000
KRAS	Sigma-Aldrich	WH0003845M1-100UG	1:1000
KRAS	Santa Cruz	Sc-30	1:1000
RAS G12D	Thermo Fisher Scientific	MA5-36256	
CD16/32	Biolegend	101302	1:200
CD45-PE-Cy7	Biolegend	147704	1:200
CD11b- FITC	Biolegend	101206	1:200
CD8-FITC	Biolegend	100706	1:200
CD11c-FITC	Biolegend	117306	1:200
CD44-FITC	BD Pharmingen	553133	1:200
CD25-FITC	Biolegend	101907	1:200
MHC-II-PE	Biolegend	107607	1:200
CD3-PE	Biolegend	100206	1:200
Ly6C-PE	Biolegend	128007	1:200
CD39-PE	Biolegend	143803	1:200
CD8-APC	Biolegend	100712	1:200
F4/80-APC	Biolegend	123116	1:200
Ly6G-APC	Biolegend	127614	1:200
CD4-APC	Biolegend	100412	1:200
NK1.1-APC	Biolegend	108710	1:200
CD86-BUV	BD Biosciences	564199	1:200
CD206-AF700	Biolegend	141734	1:200
Foxp3-AF700	Biolegend	126422	1:200
CD62L-AF700	Biolegend	104426	1:100
CD3-AF700	Biolegend	100216	1:200
